# Polystyrene-associated pulmonary fibrosis: a summary based on the available evidence and prospects

**DOI:** 10.3389/fimmu.2026.1953398

**Published:** 2026-09-28

**Authors:** Shifang Sang, Yushuai Liu, Hui Zhao, Binbin Wang, Guangchao Lv, Shiyun Feng, Kaizhong Wang

**Affiliations:** 1 Department of Thoracic Surgery, The First Hospital of Jilin University, Changchun, Jilin, China; 2 Department of Cardiac Surgery, The First Hospital of Jilin University, Changchun, Jilin, China; 3 Department of Histology and Embryology, College of Basic Medical Sciences, Jilin University, Changchun, Jilin, China; 4 Department of Urology II, The First Hospital of Jilin University, Changchun, Jilin, China

**Keywords:** ferroptosis, inflammation, inhalation toxicology, microplastics, nanoplastics, oxidative stress, polystyrene, pulmonary fibrosis

## Abstract

Microplastics have become a pervasive component of the atmosphere and of indoor and occupational air, and the human respiratory tract is now recognized as a major and largely unavoidable route of exposure. Polystyrene, one of the most abundant commodity plastics, is a dominant model and real-world airborne microplastic, and mounting evidence links inhaled polystyrene microplastics (PS-MPs) to a spectrum of pulmonary injuries that increasingly implicate fibrogenesis. This review synthesizes the current mechanistic understanding of how PS-MPs may contribute to pulmonary fibrosis, drawing primarily on preclinical experimental evidence. We first outline the sources, physicochemical determinants and respiratory deposition behavior of airborne PS-MPs, and summarize the human, epidemiological and experimental evidence for pulmonary accumulation and harm. On the basis of converging but heterogeneous evidence we propose, as a working model rather than a validated cascade, that these upstream disruptions converge on a shared profibrotic signaling hub - coupled Wnt/*β*-catenin and TGF-*β*1/Smad2/3 signaling - that orchestrates epithelial-mesenchymal transition, myofibroblast differentiation and extracellular matrix remodeling, with epigenetic and ncRNA programs stabilizing the fibrotic phenotype. Because the elements of this model rest on different levels of evidence, we grade each of them explicitly as direct PS-MP pulmonary evidence, evidence extrapolated from other particles or toxicants, or canonical fibrosis biology. No study has yet tested the complete cascade, or the causal ordering of its modules, within a single PS-MPs inhalation model. Finally, we appraise candidate therapeutic strategies, including antioxidant and ferroptosis directed approaches and repurposing of approved antifibrotics. Meanwhile, we delineate the methodological limitations including nonphysiological doses, pristi5ne monodisperse particles and short exposure windows, that must be resolved before human respiratory risk can be quantified.

## Graphical Abstract

**Figure:**
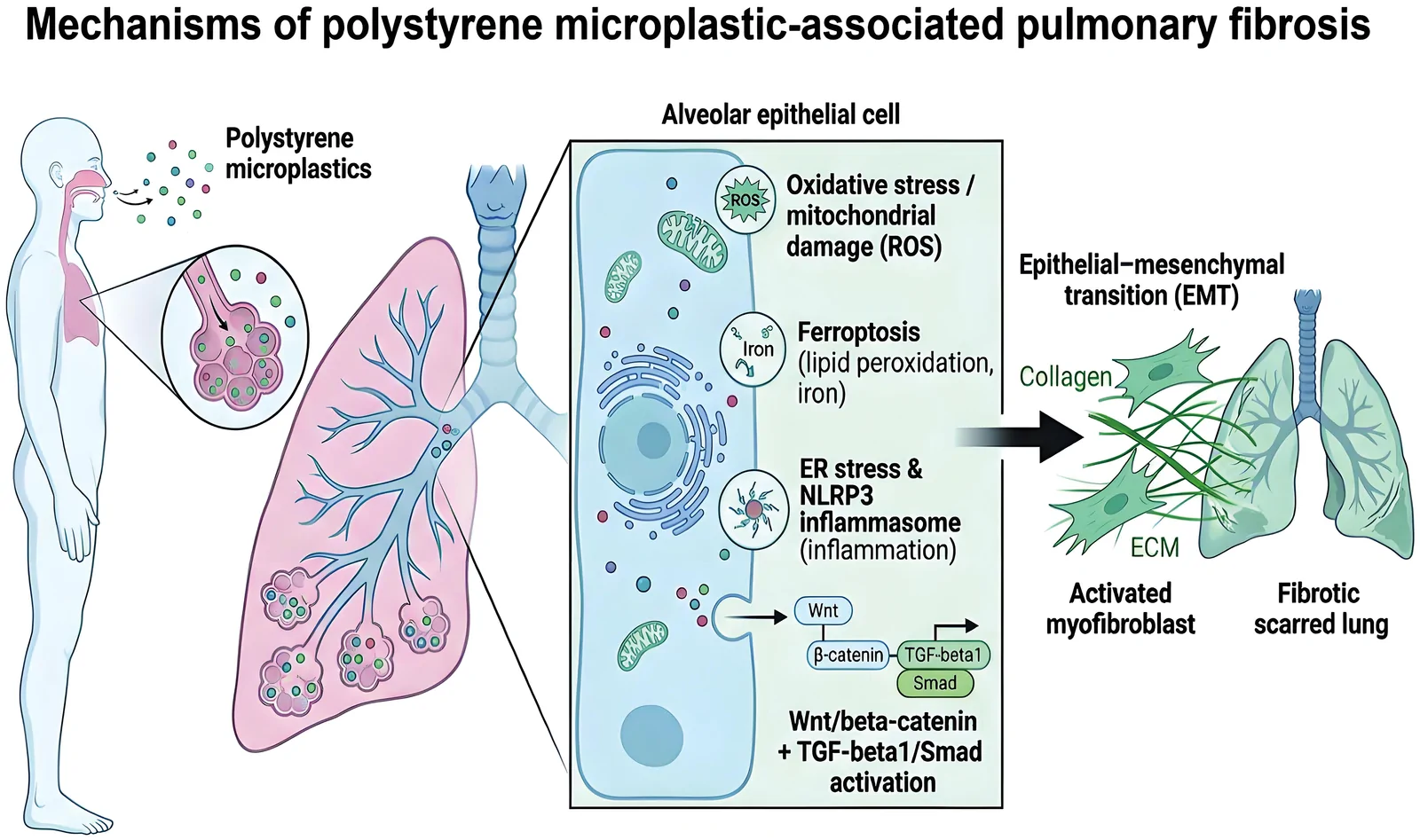
Inhaled polystyrene microplastics deposit in the distal lung, are internalized by alveolar epithelial cells, and trigger oxidative stress, ferroptosis, endoplasmic reticulum stress and inflammasome activation. These insults converge on the Wnt/*β*-catenin and TGF-*β*1/Smad signaling hub, driving epithelial–mesenchymal transition, myofibroblast activation and extracellular matrix deposition that culminate in pulmonary fibrosis.

## 1 Introduction

Global plastic production has constantly expanded since the mid-twentieth century. Cumulative output now exceeds several billion metric tons, the overwhelming majority of which has been discarded rather than recycled (1). As bulk plastics weather in the environment, mechanical abrasion, photo-oxidation and biological degradation fragment them into progressively smaller particles, generating microplastics and nanoplastics. These particles are no longer confined to marine and terrestrial matrices. They are now a quantitatively significant constituent of the atmosphere, of indoor air and of occupational environments, where they can remain airborne, undergo long-range transport and even be inhaled (2). Thus, humans are continuously exposed to particles through ingestion or inhalation, with an estimated total intake of tens of thousands per person per year (3). Because inhaled particles bypass the first-pass metabolism of the gut and can deposit directly on the vast, thin and highly vascularized alveolar surface, the respiratory tract has become the most concerned exposure route (4, 5). Report has suggested that the detection rate of microplastics in bronchoalveolar-lavage fluid in populations with suspected interstitial lung disease (ILD) is 55% and that the majority has a fibrotic phenotype. The polymers detected include polyamides, polyester, polyvinyl chloride, and polyurethane and range in size from 4.19 to 792.00 micrometers (6). In addition, fibrous microplastics account for 70 to 77% of sputum and bronchoalveolar-lavage fluid samples, and their irregular shape and small size (<100 micrometers) make them more likely to deposit and retain in the respiratory tract (7). These studies consistently show that microplastics have become a ubiquitous pollutant in the human respiratory tract, and their long-term retention and accumulation constitute a continuous stimulus source that has the potential to drive chronic inflammation and fibrotic remodeling.

Among the commodity polymers, polystyrene is especially relevant to inhalation toxicology. It is one of the most widely produced plastics and a frequent constituent of atmospheric and indoor microplastic inventories. Currently, it has become the model particle for mechanistic nanotoxicology (8, 9), because it is available commercially as well-characterized, size-defined and surface-functionalized spheres. PS-MPs are not only the real environmental pollutant, but also a research carrier that is easy to carry out experimental exploration. Therefore, the research around PS-MPs has greatly promoted the understanding of the mechanism of lung injury caused by inhalation of plastic particles.

Pulmonary fibrosis is characterized by the activation of fibroblasts, excessive deposition of extracellular matrix (ECM), and irreversible destruction of alveolar structure. The progressive decline of lung function in patients with pulmonary fibrosis represents a serious outcome that has been postulated to be linked to chronic microplastic inhalation. Idiopathic pulmonary fibrosis (IPF) and related progressive fibrosing interstitial lung diseases are characterized by relentless deposition of ECM, destruction of normal alveolar architecture, progressive loss of gas-exchange capacity and high mortality (10). Although IPF is by definition of unknown cause, its incidence has risen in many regions and shows associations with environmental and occupational particulate exposures, and fibrosis mortality clusters in industries with high inhalational dust burdens (11). The theory that inhaled, poorly soluble, biopersistent particles and fibers can drive ILD is well explained. Asbestos, silica and, notably, synthetic polymer flock provide historical precedent, such as flock worker’s lung. This is a chronic ILD-attributed to inhaled respirable nylon and other polymer fragments (12). PS-MPs share the defining hazard traits of these agents. For instance, these particles are biopersistent, exist in respirable sizes capable of reaching the distal lung and exhibit poor clearance. Therefore, it is not only biologically plausible, but also increasingly experiments support that PS-MPs inhalation could contribute to fibrogenic remodeling.

Nowadays, increasing numbers of *in vitro* and *in vivo* studies have begun to define the molecular events linking PS-MPs exposure to fibrosis in preclinical models. This literature implicates oxidative stress and mitochondrial injury, ferroptosis—an iron-dependent mode of programmed cell death characterized by lipid peroxidation— endoplasmic reticulum (ER) stress and inflammasome-driven inflammation, epithelial senescence, and activation of the canonical profibrotic transcriptional programs governed by TGF-*β*1/Smad and Wnt/*β*-catenin signaling (13–17). What has been lacking is an integrative synthesis that organizes these disparate observations into a single mechanistic frame. Overall, central thesis of this review is that the physicochemical properties of PS-MPs dictate their deposition and cellular internalization, that internalization triggers a set of parallel and mutually amplifying stress and immune responses. At the same time, these responses converge on a shared TGF-*β*1/Smad–Wnt/*β*-catenin signaling hub that executes EMT, myofibroblast activation and ECM remodeling.

## 2 Sources, physicochemical characteristics and pulmonary exposure fate of airborne PS-MPs

### 2.1 Atmospheric sources and human respiratory exposure

Airborne microplastics originate from a diverse set of sources. Primary sources include the shedding of synthetic textile fibers during wear and laundering, abrasion of tires and road markings, degradation of agricultural and packaging films, and the resuspension of plastic-laden dust. Besides, particles are also generated by the environmental fragmentation of larger plastic debris (18).Once aloft, microplastics behave as light, aerodynamically active particles capable of atmospheric suspension, wet and dry deposition, and long-range transport that delivers them even to remote regions (19, 20). Indoor air is frequently more heavily contaminated than outdoor air because of dense concentrations of synthetic furnishings, carpets and clothing in poorly ventilated spaces, which is significant given the large fraction of time humans spend indoors. Integrating dietary, drinking-water and inhalational pathways, human microplastic intake has been estimated to reach tens of thousands of particles annually, with inhalation contributing a substantial and possibly dominant share for the finest, most respirable fractions (3). Combined with the above exposure assessment data and the direct detection evidence of microplastics in human respiratory tissues, it can be confirmed that inhalation is the real route of continuous entry of PS-MPs into the human body.

Several features of respiratory exposure distinguish it toxicologically from ingestion. First, the alveolar epithelium presents an enormous surface area that is only a fraction of a micrometer thick and is intimately apposed to the pulmonary capillary bed, so deposited particles can diffuse into systemic blood circulation easily. Second, unlike the gut, the distal lung lacks a robust mucociliary escalator, clearance of alveolar particles depends chiefly on comparatively slow macrophage mediated phagocytosis and lymphatic drainage, which are readily overwhelmed by biopersistent material, favoring long retention (21, 22). Third, occupational and indoor microenvironments can generate localized airborne concentrations far exceeding ambient outdoor levels, so exposure is highly heterogeneous across individuals and settings (2, 18). These features together suggest that even moderate concentrations of airborne transmission, sustained over many years, can confer considerable cumulative alveolar doses. Precisely, the chronic and low-dose exposure pattern is most relevant to slowly developing fibrotic disease, but least well captured by current acute experimental models.

### 2.2 Physicochemical determinants of hazard: size, shape and surface charge

The biological behavior of PS-MPs is governed less by polymer identity alone than by an ensemble of physicochemical properties, including particle size, shape, surface charge, surface chemistry and the adsorbed molecular corona, that together determine where particles deposit, whether and how they are internalized, and how vigorously they perturb cell physiology (9, 23). Size is the major variable for the respiratory tract. Aerodynamic diameter dictates the depth of deposition. Larger particles are intercepted in the upper airways and cleared by mucociliary transport, particles in the low-micron range deposit in the conducting and small airways, and the finest micro/nanoscale particles, penetrate to the alveoli, where clearance is slow and residence times are long (24). Smaller particles however, possess relatively large surface-area-to-mass ratios, amplifying surface-mediated reactivity and adsorption. Consistent with this, smaller polystyrene particles are internalized more readily and are more cytotoxic to lung cells than larger counterparts across multiple *in vitro* systems (25–27). But that’s not representative either. In a chronic inhalation experiment, 1 μm microplastics induced more severe pulmonary toxicity than 80 nm nanoparticles, with stronger effects on promoting epithelial-mesenchymal transition and fibrosis, suggesting that particle size toxicity is not a simple inverse correlation, and its complexity may involve different cellular uptake and clearance kinetics (28). Another study compared the inhalation effects of micron-scale (mps) and nano-scale (nps) polystyrene and found that mps group had a more significant reduction in lung volume, decreased exercise capacity and enhanced EGFR expression, indicating that under certain conditions, micron-scale particles are more pathogenic than nano-scale particles (29).

Surface charge is a second critical determinant. Comparative studies of aminated (positively charged) versus carboxylated (negatively charged) polystyrene nanoparticles demonstrate that cellular uptake, intracellular trafficking and toxicity are strongly charge-dependent, with cationic particles typically internalized more efficiently and provoking more significant ROS production and mitochondrial damage (30–33). Importantly, the biomolecular corona, a layer of proteins and lipids that rapidly adsorbs onto particle surfaces in biological fluids, could reshape the effective surface presented to cells and can dominate the particle–cell interaction, a phenomenon detailed at the biophysical level for polystyrene particles interacting with biomolecules and model membranes (34, 35).

A crucial caveat is that PS-MPs are not single or reproducible entity. Nominally identical commercial particles from different suppliers, or even different batches, can differ markedly in their residual additives, surfactants, leachates and surface state, and these differences may contribute to substantial variability in particle–cell interactions and toxicity (34, 36). This heterogeneity is a certain source of the apparent inconsistencies across studies and a central methodological challenge.

### 2.3 Deposition, internalization and biological fate in the lung

Following deposition in the distal lung, PS-MPs encounter alveolar epithelial cells (chiefly type I and type II pneumocytes) and resident alveolar macrophages. Uptake proceeds primarily through energy-dependent endocytic routes, including clathrin and caveolae mediated endocytosis and macropinocytosis followed by endolysosomal trafficking. Studies tracking polystyrene document their endocytosis, intracellular distribution and comparatively inefficient exocytosis, which favors intracellular accumulation (8, 31). Persistent particles that accumulate in lysosomes can impair lysosomal function, destabilize membranes and seed downstream stress responses (37). Because polystyrene is biopersistent and not enzymatically degraded, particles are retained, and the smallest fractions may translocate across the air–blood barrier into the systemic circulation, consistent with the detection of plastic particles in human blood and distant organs (38). The reliable analytical detection and quantification of these particles in complex biological matrices remains difficult. However, advances in pyrolysis–gas chromatography/mass spectrometry and related mass-based methods are improving the rigor of exposure assessment (39).

The kinetics of these processes are central to fibrogenic risk. Because polystyrene is not biodegradable and alveolar clearance is slow, the major variable is the balance between the rate of deposition and the rate of removal. When deposition outpaces macrophage mediated clearance—as is likely under chronic exposure or with impaired clearance in lung—particles accumulate. It is the retained long-resident burden rather than any single exposure event that is expected to drive disease. Two consequences follow. First, particle size exerts a dual influence. Smaller particles deposit more distally and are internalized more readily, but the very smallest may also translocate out of the lung into the circulation, redistributing rather than accumulating locally, whereas mid-range respirable particles may be optimally retained in the alveolar region (40). Furthermore, pharmacokinetic studies showed that the nanoparticles were too small in aerodynamic size to achieve ideal deposition during direct lung delivery (41). The size fraction of greatest fibrogenic concern is therefore not simply volume and remains to be defined empirically. Second, uptake by alveolar epithelial cells themselves is particularly consequential for fibrosis, because it places the particle-induced stress cascade directly within the cells whose injury, death and senescence initiate remodeling (26, 31). **Figure 1** summarizes the source-to-cell trajectory of airborne polystyrene.

**Figure 1:**
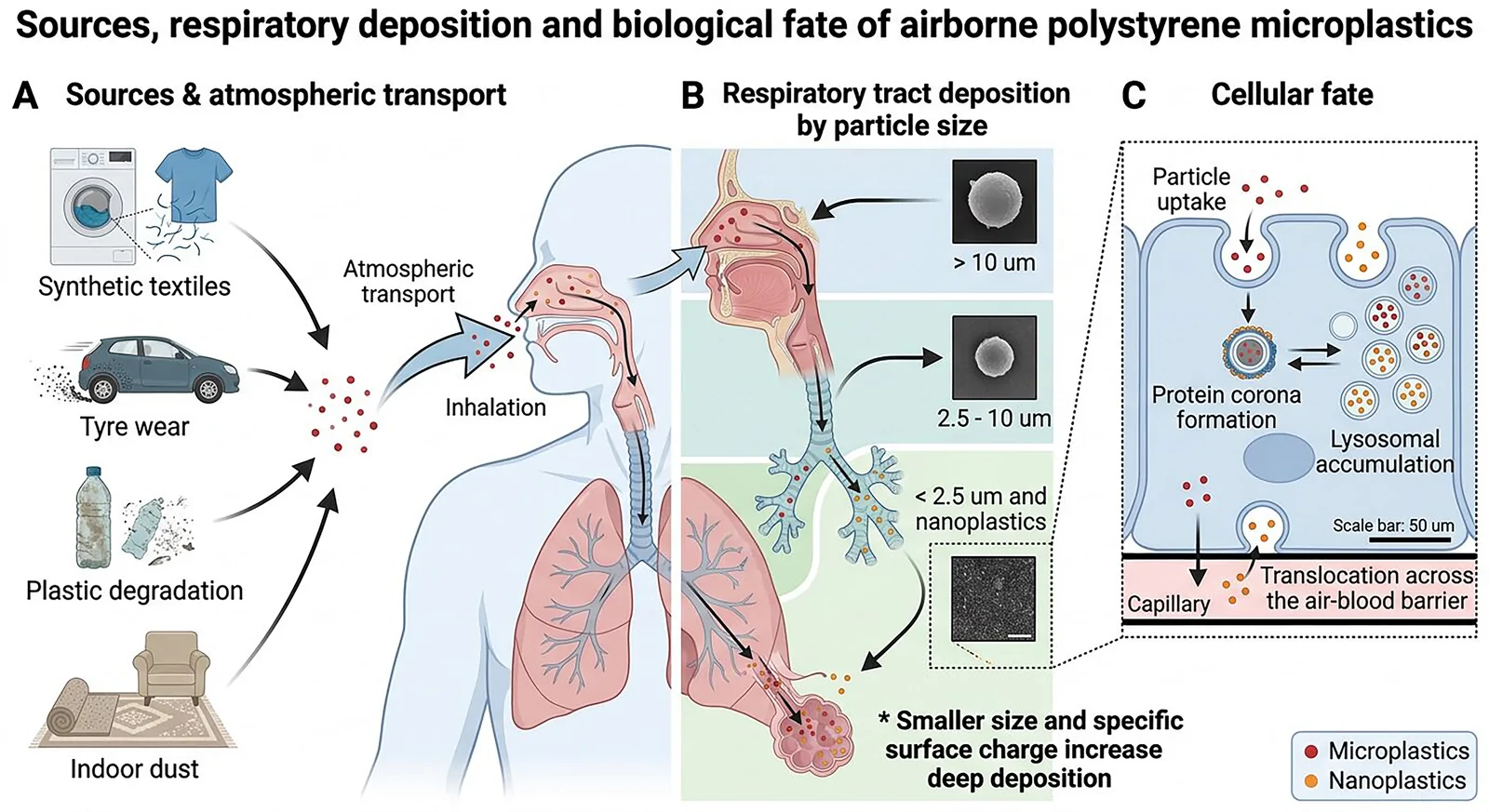
Sources, respiratory deposition and biological fate of airborne polystyrene microplastics. Primary and secondary emissions loft microplastics into indoor and outdoor air **(A)**. Aerodynamic diameter governs the depth of deposition along the respiratory tree, with the finest fractions reaching the alveoli **(B)**. Deposited particles are internalized by alveolar epithelial cells via endocytosis, acquire a biomolecular corona, accumulate in lysosomes and may eventually translocate across the air–blood barrier **(C)**. Importantly, no study has yet established a causal, dose-resolved epidemiological link between ambient polystyrene exposure and incident pulmonary fibrosis. The human evidence to date establishes exposure and biological plausibility rather than causation.

## 3 Harm and epidemiological evidence for pulmonary exposure

The strongest justification for concern about inhaled PS-MPs are the direct demonstration that microplastics reach and reside within the human respiratory system. Amato-Lourenço and colleagues identified microplastic particles and fibers in human lung parenchyma obtained at autopsy, establishing that inhaled plastics penetrate to and persist in deep lung tissue (42). This finding has since been extended and corroborated. Microplastics have been detected across the length of the human respiratory tract (38, 43), and their presence in lung tissue has been associated with clinical and laboratory indices in patients, suggesting that particle burden is not merely a passive marker of exposure but may track with physiological perturbation (44). Independent work has reported micro/nanoplastics in bronchoalveolar and other respiratory specimens, reinforcing those particles reach the alveolar compartment where fibrogenesis originates (45). Beyond the lung, microplastics have been quantified in human blood, confirming systemic bioavailability of at least the smaller fractions and raising the possibility of extrapulmonary and cardiovascular consequences of respiratory exposure (46).

Two major challenges accompany such human tissue measurements. First, quantifying microplastics, particularly nanoplastics, within tissues is technically demanding. Measurements are prone to procedural contamination and heavily method-dependent, leading to substantial uncertainty in absolute burden estimates and poor cross-study comparability. Mass spectrometry-based techniques such as pyrolysis-gas chromatography/mass spectrometry have improved analytical accuracy yet fail to detect the smallest particles (39). Second, correlations between pulmonary microplastic loads and clinical/laboratory are consistent with a causal relationship but cannot prove it, as Particle accumulation may also be the result of impaired clearance of diseased lung tissue (44). Despite the above limitations, the convergence of tissue-level quantification, systemic bioavailability data and coherent mechanistic evidence transforms inhalable PS-MPs from a putative respiratory hazard to a relative plausible one.

Systematic review of a large number of relevant literatures further improved the above basic detection evidence. A systematic review on microplastics and nanoplastics and human respiratory health pointed out that although this research field started late and the detection methods of various studies are quite different, plastic particles are continuously detected in human respiratory tissues, and there is a complete and reasonable pathogenic mechanism as support. Therefore, inhaled microplastics should be regarded as a new type of harmful respiratory pollutants (47). All kinds of narrative reviews and mechanism reviews also consistently emphasized that plastic and microplastic pollution in the air can produce quantifiable and biologically significant damage effects on respiratory diseases, and pointed out that there was still a significant gap in the current human related research data (4, 5). Reviews focused on vulnerable populations note that individuals with pre-existing chronic lung disease may be disproportionately affected, and that microplastics may act as co-factors in respiratory carcinogenesis and disease progression (48, 49).

However, these cross-sectional studies, as well as review articles, have ignored the influence of confounding factors. These confounding factors mainly include smoking, occupational exposure and underlying lung diseases. In theory, they can affect the deposition of microplastics in the lung and the occurrence and development of pulmonary fibrosis. From a pathophysiological standpoint, these factors independently impair pulmonary clearance of inhaled microplastics and thereby create conditions that favor their subsequent profibrotic effects. Cigarette smoking disrupts mucociliary clearance through ciliary shortening and reduced beat frequency, while simultaneously impairing alveolar macrophage phagocytic and bactericidal activity. smoke also augments oxidative stress and compromises epithelial barrier integrity, collectively facilitating particle retention in the lung interstitium. Notably, smokers have been shown to retain more inhaled particles than non-smokers due to overwhelmed macrophage-mediated clearance pathways (50). Chronic occupational exposure to mineral dusts, fibers, or chemical fumes frequently induces macrophage dysfunction, impaired lymphatic drainage, and varying degrees of structural remodeling, establishing a microenvironment that may promote microplastic deposition and sustains chronic foreign-body reactions. Occupations involving plastic manufacturing, textiles, construction, and mining carry particularly elevated ambient microplastic concentrations, yet these same environments harbor established fibrogenic inorganic dusts — such as crystalline silica and asbestos — that independently drive pneumoconiosis and hypersensitivity pneumonitis (51). Pre-existing chronic lung diseases, including COPD, bronchiectasis, and idiopathic pulmonary fibrosis, are themselves characterized by impaired clearance, abnormal airway architecture, and persistent inflammation–repair imbalance, which predispose to microplastic accumulation in diseased regions and subsequent fibroblast activation. Consistent with this, PS-MP concentrations are significantly elevated in lung tissues of COPD patients compared with controls, and microplastics have been detected in bronchoalveolar lavage fluid from a substantial proportion of patients with suspected interstitial lung disease, particularly those with fibrotic phenotypes (52). Asthmatic and COPD epithelial cells also exhibit heightened susceptibility to microplastic-induced damage compared with healthy controls (53). Collectively, although direct human evidence remains limited, it is reasonable to infer that these three factor categories not only independently increase the risk of pulmonary microplastic retention and fibrotic progression, and may also exert additive or synergistic effects when coexisting. Critically, because each of these factors influences both particle retention and fibrosis progression, they constitute substantial confounders in cross-sectional association studies. Future studies need to shift from PS-MPs exposure alone to real-world complex co-exposure. Prospective cohort or case-control studies were conducted in epidemiology, adjusting for confounding factors such as smoking, occupational exposure, and basic lung diseases.

## 4 Experimental models and methodological considerations

Mechanistic knowledge of PS-MPs pulmonary toxicity derives from a series of experimental systems. *In vitro* studies predominantly employ human alveolar epithelial cell lines (notably A549 and BEAS-2B), primary or immortalized bronchial epithelium, and macrophage models, exposing them to size-defined and charge-defined polystyrene spheres (26, 27, 54). Such *in vitro* systems can accurately elucidate particle uptake, oxidative stress, inflammasome activation, and cytotoxicity in dose, particle size, and charge-dependent ways, but cannot reconstruct the multicellular structure, mechanical dynamic characteristics, and particle clearance process of the intact lung. *In vivo* studies about fibrosis, principally in rodents, use intratracheal instillation, oropharyngeal aspiration, or inhalation to deliver PS-MPs and assess histopathology, collagen deposition and molecular signaling over days to months (14, 16, 55, 56). Instillation delivers a precisely known dose but bypasses the physiological filtering (57) and regional deposition of true inhalation and can induce acute inflammation and structural destruction (58). Whole-body or nose-only inhalation is more realistic but technically demanding and dosimetrically complex.

Three methodological issues recur across this literature and temper its interpretation. First, the dose and dose rate used in the experiments are usually much higher than the real-world exposure level: a large number of studies use acute exposure, the exposure dose can reach the milligram order, and the exposure concentration is micromolar to millimolar. However, real human inhalation exposure belongs to long-term and low-dose exposure, so the conclusion that extrapolates the experimental toxicity risk to the environmental human exposure risk is highly uncertain. Second, most studies have used commercially available standard particles, which are primitive, monodisperse, spherical, and with well-defined components, which cannot truly represent microplastics in the environment. After long-term aging, environmental microplastics have irregular morphology and heterogeneous composition, and their surface is also coated with organic matter canopy. Particle aging will change its surface chemical properties and pollutant adsorption capacity, and then change its self-toxicity (36, 59). Third, the biological identity of PS-MPs is not standardized. As noted above, nominally identical particles differ across suppliers and batches, and residual surfactants or endotoxin contamination can confound apparent particle effects (34, 36). Particle toxicology may help calibrate the relative potency of plastic particles and situate them within established inhalation hazard frameworks (60). **Table 1** summarizes representative experimental models, exposure parameters and principal fibrosis relevant endpoints.

**Table 1:** Representative experimental models of polystyrene (and related) microplastic pulmonary toxicity, exposure parameters and fibrosis-relevant endpoints.

Model/system	Particle & exposure	Principal fibrosis-relevant Findings	References
Mouse intratracheal instillation	PS-MPs repeated instillation	Collagen deposition activation of Wnt/*β*-catenin oxidative stress fibrotic remodeling	(14)
Rat intratracheal instillation	PS-MPs/NPs	TGF-*β*1/Smad activation EMT pulmonary fibrosis	(16)
BEAS-2B	PS-NPs	Lung injury via inflammatory oxidative pathways	(61)
Mouse, inhalation	Tire-wear microplastic particles	Pulmonary fibrotic injury after inhalation	(55)
Mouse intratracheal instillation	PS, PP, PET particles	Comparative pulmonary inflammation and toxicity by polymer	(62)
Chicken	PS-MPs	Endoplasmic reticulum stress–driven NLRP3	(63)
A549 lung epithelium (*in vitro*)	PS-MPs/NPs	Uptake, ROS, cytotoxicity, bioenergetic impairment	(26, 27, 64)
Macrophages (*in vitro*)	PS-MPs	Immunometabolic reprogramming	(54)
Lung epithelium (*in vitro*/*in vivo*) and mouse	PS-NPs	YY1/FTL-dependent ferroptosis fibrosis	(17)
Rat lung/epithelium	PS-MPs	cGAS–STING–mediated ferroptosis and fibrosis	(15)

Doses are as reported in the primary studies.

The dosimetry problem deserves particular scrutiny. Meaningful extrapolation requires translating an administered mass or particle number into a biologically relevant metric—ideally the surface area of particles deposited per unit of alveolar epithelium, integrated over time (65). Most studies report neither deposited dose nor surface-area dose, and acute bolus instillation of milligram quantities can transiently expose alveolar cells to particle concentrations that chronic ambient inhalation might approach only over years or never. Bolus delivery also produces agglomeration and uneven regional distribution that do not occur with inhalational deposition, potentially generating artefactual local overload responses (66, 67). Consequently, a mechanism robustly demonstrated at high dose (for example, ferroptosis or inflammasome activation) may operate quite differently, or not at all, at environmentally realistic doses and dose-rates. This does not negate the conclusions of existing mechanistic studies. However, the current field can partly confirm the hazard potential and potential pathogenic pathways, but it is difficult to reliably confirm the health risk under real environmental exposure.

## 5 Core molecular mechanisms of PS-MPs induced pulmonary fibrosis

The existing preclinical research evidence can be organized into a coherent working model of pathogenic cascades. It begins with particle internalization, which initiates multiple parallel stress and immune pathways. These signals then converge on shared profibrotic signaling hubs. This model is based on converging evidence from disparate experimental systems and should be regarded as hypothesis-generating rather than a fully validated causal chain. The overall process is shown in **Figure 2**.

**Figure 2:**
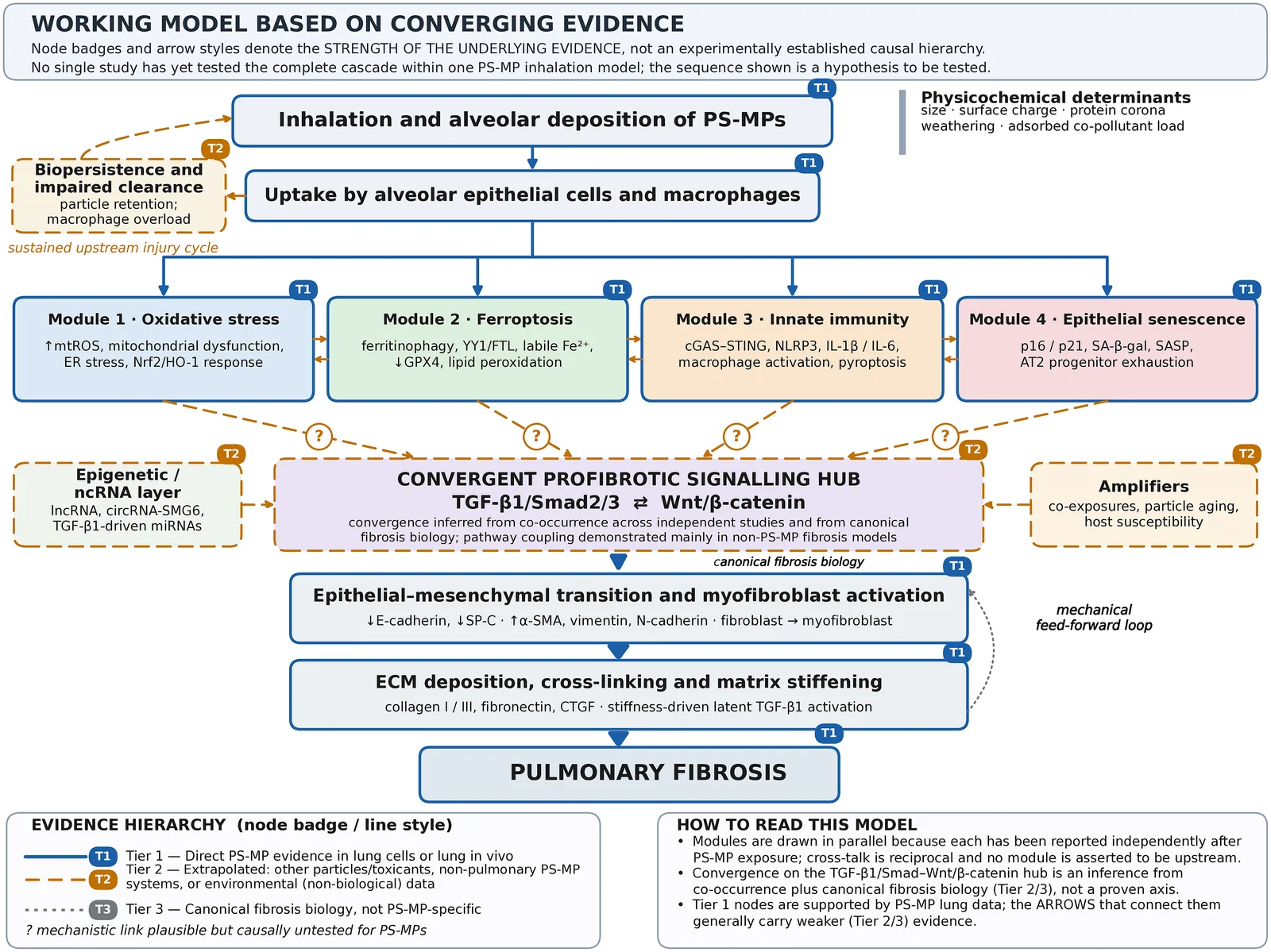
A framework of PS-MPs-associated pulmonary fibrogenesis based on converging evidence. Inhaled PS-MPs deposit in the distal lung and are internalized by alveolar epithelial cells and macrophages. Because polystyrene is not biodegradable and alveolar clearance is slow, retained particles constitute a sustained upstream injury source. Four injury modules — oxidative stress with mitochondrial and endoplasmic-reticulum dysfunction, ferroptosis, innate immune activation, and epithelial senescence with a senescence-associated secretory phenotype — have each been reported independently after PS-MPs exposure. The reciprocal dotted arrows denote cross-talk of undetermined order. These modules are proposed to converge on coupled TGF-*β*1/Smad2/3 and Wnt/*β*-catenin signaling, which in turn drives epithelial-mesenchymal transition, myofibroblast activation, extracellular matrix deposition and matrix stiffening. Evidence explanation: node badges and line styles indicate Tier 1 (direct PS-MPs evidence in lung cells or lung *in vivo*, solid blue), Tier 2 (evidence extrapolated from other particles or toxicants, from non-pulmonary PS-MPs systems, or from environmental non-biological data, dashed amber) and Tier 3 (canonical fibrosis biology that is not PS-MPs-specific, dotted gray). Circled question marks denote mechanistically plausible linkages that remain causally unvalidated under PS-MPs exposure conditions. To date, no single inhalation study has experimentally validated this full pathological cascade. The illustrated pathway represents a testable mechanistic hypothesis for future investigations.

### 5.1 Oxidative stress and mitochondrial dysfunction

After internalization of PS-MPs by cells, the most commonly reported early effect is a massive burst of ROS, accompanied by mitochondrial dysfunction. *In vitro* models of alveolar epithelial cells and macrophages, polystyrene particles can increase intracellular and mitochondrial ROS levels, consume reduced glutathione, damage mitochondrial membrane potential, inhibit mitochondrial respiration, and eventually cause energy metabolism failure in cells (27, 64, 68). Impaired mitochondrial electron transport chain leads to electron leakage and becomes the main source of ROS, which in turn attacks mitochondrial membrane structures and further aggravates mitochondrial damage, ultimately driving lung epithelial cells and macrophages into a profibrotic phenotype (69, 70).

Cells will initiate a compensatory defense response through the Nrf2/HO-1 antioxidant pathway. The balance between the level of ROS production and the ability of Nrf2 mediated antioxidant defense is a central factor in determining epithelial cell survival, senescence, or death. If the Nrf2 signaling pathway is insufficiently activated or functionally exhausted, cell damage will continue to be aggravated (71). At present, it is generally believed that Nrf2 is not only a key regulatory node in the pathogenesis of pulmonary fibrosis, but also a potential therapeutic target (72). Experimental interventions that bolster mitochondrial antioxidant capacity mitigate particle-induced oxidative injury, underscoring the causal role of mitochondrial ROS (73). In addition, oxidative stress can also be mediated by the up-regulation of enzymes such as NOX4, and knockdown of NOX4 gene reversed the cellular effects induced by PS-NPs, confirming its key role in cell damage (74).

Excess ROS oxidize lipids, proteins, and DNA, and act as upstream initial signals to initiate multiple downstream pathways, including inflammasome activation and programmed cell death (15, 75, 76). Oxidative stress rapidly triggers the downstream inflammatory cascade. Exposure to PS-MPs resulted in significant inflammatory cell infiltration in lung tissue and increased release of pro-inflammatory factors such as IL-6, TNF-α and IL-1*β* (68, 77). In bronchoalveolar lavage fluid, neutrophil counts can be increased 3.9-fold by UV-aged fragmented nanoplastics exposure, and the intensity of the inflammatory response is positively correlated with the intrinsic oxidative potential of the particles (75, 78). The key regulatory hub of this inflammatory response is the NLRP3 inflammasome. Both PS-MPs and PS-NPs could effectively activate NLRP3 inflammasome and promote the activation of caspase-1 and the release of IL-1*β* (75, 79). Particle size dependence is a significant feature of PS-MPs toxicity, with smaller size particles inducing more severe lung injury and inflammatory response (80, 81). Mechanistically, PS-MPs further amplify inflammatory signals by activating NF-κB and other signaling pathways. For example, small size PS-MPs can activate NF-κB signaling by targeting TLR2, and inhibition of this pathway can alleviate inflammation, oxidative stress, and control fibrosis progression (82). In addition, endoplasmic reticulum stress, metabolic disorders (such as arachidonic acid metabolism pathway) and dysfunction of circadian gene Bmal1 are also involved in PS-MPs induced lung inflammation (71, 77, 83). Lipid peroxidation is a widely reported early event in lung injury models exposed to polystyrene particles. For example, exposure to PS-NPs resulted in decreased ferroptosis key proteins GPX4 and FTH1 in lung epithelial cells, accompanied by increased levels of malondialdehyde (MDA) (84). *In vivo*, inhalation of 100 nm amino-modified polystyrene nanoplastics (APS-NPs) also induced oxidative stress and ferroptosis in lung tissues of mice (71). Besides, persistent oxidative DNA damage activates the p53-p21 pathway and induces cellular senescence (85). Thus, oxidative stress should not be viewed as an injury terminal event but rather as an initial amplified signal upstream of the pathway that progressively drives ferroptosis, inflammasome activation, cellular senescence. Oxidative stress is at the upstream of the whole injury cascade, which is the core reason why targeted antioxidant and Nrf2 intervention strategies have research value.

### 5.2 Ferroptosis

A major breakthrough in the field of mechanism research is that polystyrene (mainly nanoparticles) can induce ferroptosis in lung epithelial cells, a kind of programmed cell death mediated by iron ion dependence and lipid peroxidation. Ferroptosis is closely related to pulmonary fibrosis. The occurrence of ferroptosis is characterized by the accumulation of oxidized iron and the inactivation of glutathione peroxidase 4 (GPX4) antioxidant defense system, which together lead to uncontrolled peroxidation damage of cell membrane phospholipids. A number of studies have confirmed that ferroptosis is the core link of pulmonary epithelial cell injury caused by polystyrene. PS-NPs can induce ferroptosis in lung epithelial cells, accompanied by the hallmark lipid peroxidation, intracellular iron accumulation and the inhibition of GPX4 expression/activity. Drug specific blockade of ferroptosis pathway can significantly alleviate the above cell damage (86). Mechanistically, several converging axes have been defined. First, oxidative stress induced by PS-NPs can activate ferroautophagy — a selective form of autophagy whereby ferritin is degraded to liberate free intracellular iron — which further accelerates the Fenton reaction and promotes lipid peroxidation (87). Second, PS-NPs engage the transcription factor YY1 to upregulate ferritin light chain (FTL), remodeling cellular iron handling in a manner that promotes ferroptosis and downstream lung epithelial fibrogenesis (17). Third, the cytosolic DNA sensor cGAS and its adaptor STING—classically an innate immune pathway responsive to mitochondrial and nuclear DNA released during oxidative injury—have been shown to mediate PS-MPs induced ferroptosis and pulmonary fibrosis, linking particle-triggered mitochondrial damage to both innate immune activation and ferroptosis (15).

The broader relevance of ferroptosis to fibrogenesis is well supported. Ferroptosis of alveolar epithelial cells is emerging as a driver of pulmonary fibrosis generally, is mechanistically tied to EMT, and also represents an actionable therapeutic node (88–90). The cGAS–STING axis likewise has an established profibrotic role in the lung independent of plastics, reinforcing the plausibility of this mechanism (91). Ferroptosis inhibitors (such as Fer-1 and deferoxamine) can effectively alleviate pulmonary epithelial injury and fibrosis- like pathology induced by PS-NPs, which were visualized and verified in the lung microarray model (87).

It is worth highlighting that lipid peroxidation products (LPOs) are not only the terminal executors of ferroptosis, but also may act as upstream signals to directly trigger the assembly and activation of NLRP3 inflammasome. Particulate matter (PM) induced lipid peroxidation, iron accumulation, and ROS production, on the one hand, are characterized by ferroptosis, on the other hand, they trigger the excessive activation of inflammasome, leading to inflammation mediated cell death (92). This finding reveals that the iron-ROS axis is a key mechanism linking ferroptosis to inflammasome activation. In the study of sepsis-induced acute lung injury, RBA nanoparticles inhibited lipid peroxidation-mediated ferroptosis by restoring redox homeostasis, while inhibiting NLRP3 inflammasome assembly by directly binding to its NACHT domain (93). In monocytes from severe COVID-19 patients, the activation of NLRP3 inflammasome is strongly correlated with the abnormal increase of mitochondrial superoxide and lipid peroxidation levels, and the secretion of IL-1*β* is partially dependent on lipid peroxidation (94). These evidences suggest that lipid peroxidation induced by polystyrene particles not only drives ferroptosis, but also produces active lipid peroxides (such as MDA and 4-HNE), which may serve as damage-associated molecular patterns (DAMPs) and directly provide platform signals for NLRP3 assembly, thus forming the primary molecular bridge for ferroptosis-driven inflammasome activation.

Ferroptosis of alveolar type II epithelial cells does not merely deplete the regenerative epithelial pool—itself a profibrotic event because failed re-epithelialization favors fibroblast dominance—but actively feeds the inflammatory and TGF-*β*1-rich milieu that drives remodeling. Simultaneously, Ferroptosis is not only a terminal event of cell death, but its products, such as 4-hydroxynonenal (4-HNE) and other lipid peroxidation derivatives, can directly up-regulate TGF-*β* expression and promote fibrosis signaling (95). The mechanistic linkage between epithelial ferroptosis and EMT has been demonstrated directly (88), positioning ferroptosis as both a death program and a signaling event that hands off to the profibrotic hub. **Figure 3** details this cascade.

**Figure 3:**
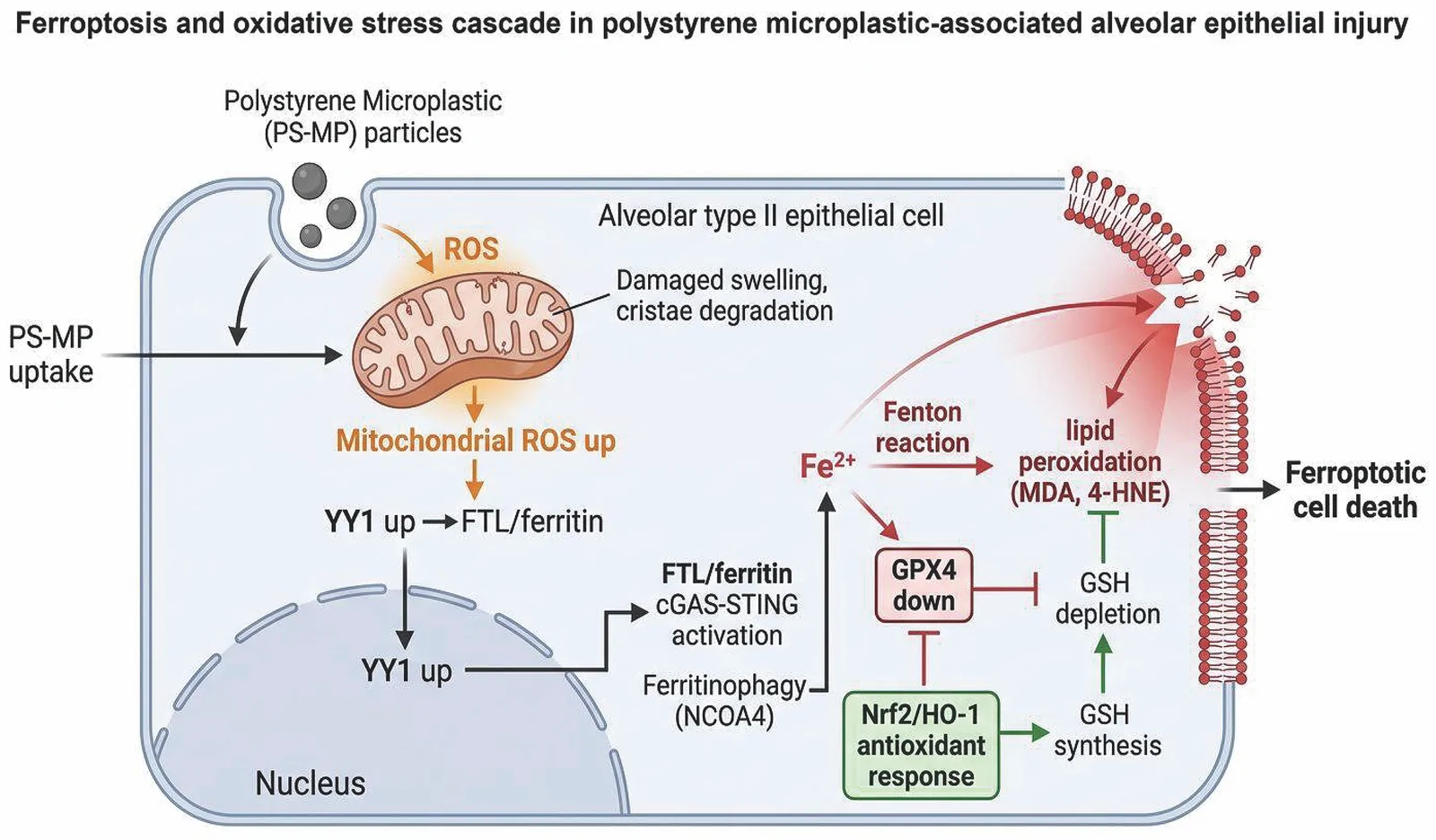
Ferroptosis and oxidative-stress cascade in PS-MPs-injured alveolar epithelial cells. Internalized particles generate mitochondrial ROS, which activates cGAS–STING and the YY1-FTL axis and drives ferritinophagy. Liberated Fe^2+^ fuels Fenton reaction and lipid peroxidation, while GPX4 suppression and glutathione depletion remove antioxidant defenses, culminating in ferroptosis. The Nrf2/HO-1 response provides protection.

Apoptosis and ferroptosis have a synergistic effect in destroying alveolar results and promoting fibrosis. Apoptosis has long been considered to be an important expression of pulmonary toxicity of PS-MPs. Polystyrene exposure can induce apoptosis of alveolar epithelial cells by activating caspase-3 through oxidative stress and mitochondrial pathways. Nasal exposure of PS-MPs (1-5 μm) can up-regulate TLR2 and activate NF-κB signaling pathway, promote oxidative stress and apoptosis, and lead to collagen deposition and fibrosis (82). Apoptosis can lead to loss of cell membrane integrity, release of intracellular contents and double-stranded DNA, activation of the cGAS-STING signaling pathway, and further amplification of inflammatory and fibrotic responses (96). In addition, in the physiological structure, the massive apoptosis of alveolar epithelial cells directly causes the rupture of alveolar septum and the collapse of alveolar space (97), which provides space for fibroblast proliferation and extracellular matrix deposition, and interacts with inflammatory response to aggravate the fibrosis process (98).

### 5.3 Innate immune activation and inflammation

Pulmonary fibrosis induced by PS-MPs in experimental models is not a single process of matrix deposition, but a regulatory network involving immune cells, inflammatory mediators and fibrotic pathways. In the lung specifically, inhalation exposure of PS-MPs can cause severe inflammatory response in the lungs of mice, which is manifested as increased myeloperoxidase (MPO) and inflammatory cell infiltration (80, 82). Small-size PS-MPs can bind to Toll-like receptor 2 (TLR2) and activate the NF-κB signaling pathway, thereby aggravating inflammation, oxidative stress, and finally promoting the process of pulmonary fibrosis (82). Inflammatory cell infiltration is an important accompanying feature of the progression of pulmonary fibrosis. At the level of immune cells, alveolar macrophages are the core response cells for the pulmonary toxicity of polystyrene particles. PS-NPs can activate the unfolded protein response (UPR)/oxidative stress/IL-6 signaling axis of alveolar macrophages, induce oxidative stress-dependent IL-6 secretion, and promote the transformation of fibroblasts into myofibroblasts, thereby driving pulmonary fibrosis. IL-6 deficiency or neutralization can significantly inhibit fibrosis (69). In addition, the combined exposure of PS-NPs and benzo (a) pyrene can trigger the formation of macrophage extracellular traps (METs) through PI3K-AKT and MAPK cascades, which aggravates alveolar inflammation and pulmonary fibrosis (99). At the level of adaptive immunity, PS-MPs inhalation exposure can mediate Th2 immune response through IL-33 signaling pathway, leading to asthma-like symptoms, including airway inflammation and hyperresponsiveness. Epithelial cells play a key driving role in this process (100).

A number of *in vivo* comparative studies have confirmed that polystyrene particles and other polymer particles can induce significant lung inflammation, and the intensity of inflammation depends on the type of polymer, particle size and exposure dose (56, 62). At the heart of this response is the NLRP3 inflammasome, a cytosolic multiprotein complex (75). When activated, it can promote the maturation of interleukin-1*β* (IL-1*β*) and TGF-*β* and induce pyroptosis—an inflammatory programmed cell death mediated by activation of caspase-1 (or caspase-4/5/11) by inflammasome. These factors not only amplify the local inflammatory response, but also act on alveolar epithelial cells and fibroblasts in a paracrine manner to reshape the profibrotic microenvironment (101). Microplastics and nanoplastics can act as danger signals to activate NLRP3. It has been proposed that inflammasomes can be used as universal sensors for various types of cells to sense plastic particle exposure (102).

At the mechanistic level, mitochondrial dysfunction is closely related to NLRP3 inflammasome activation. Damaged mitochondria are not only the main source of ROS, but also the key organelles that release damage-associated molecular patterns (DAMPs). Mitochondrial dysfunction leads to the release of mitochondrial DNA (mtDNA) into the cytoplasm, which is one of the classical pathways to activate the NLRP3 inflammasome. In both *in vitro* and *in vivo* models of nanoplastic exposure, significant oxidative stress, cell death, and inflammation have been observed, accompanied by excessive release of double-stranded DNA (dsDNA), which activates the cGAS-STING signaling pathway and promotes pulmonary fibrosis (96). ATG16L1 deficiency in macrophages leads to the accumulation of mitochondrial ROS, which activates NLRP3 excessively through the cGAS-STING signaling pathway and exacerbates alveolar epithelial cell damage (103). PS-MPs induced mitochondrial dysfunction and mito-ROS accumulation can be alleviated by mitochondria-specific ROS scavengers or ferroptosis inhibitors, thereby reducing excessive inflammation and improving acute exacerbations of COPD (104). Persistent chronic inflammation is a recognized precursor event of tissue fibrosis and remodeling, which continuously creates a cytokine-rich microenvironment to promote myofibroblast differentiation and ECM accumulation. The continuous role of IL-6 and TGF-*β*1 is particularly critical in this process.

In the PS-MPs exposure model, evidence has confirmed that it can induce alveolar epithelial ferroptosis, and drive the activation of NLRP3 inflammasome and the release of pro-inflammatory factors. However, it has not been tested whether there is a regulatory relationship between ferroptosis and NLRP3 inflammasome under PS-MPs injury conditions. Polystyrene particles exposure significantly activates the NLRP3 inflammasome and promotes the release of IL-1*β*. In other toxic exposure systems, such as cadmium-induced lung injury model, ferroptosis inhibitor Fer-1 can reduce the expression of NLRP3 and ASC, inhibit caspase-1 activation, and reduce the production of IL-1*β* and IL-18, suggesting a close regulatory relationship between ferroptosis and NLRP3 inflammasome activation (105). At the transcriptional level, YAP1 knockdown leads to increased levels of pro-inflammatory factors, accompanied by decreased SLC7A11 expression and increased lipid peroxidation in PM2.5-induced lung injury (106), suggesting that inflammatory factors may regulate cellular sensitivity to ferroptosis by affecting the expression of the Yap1-SLC7A11 axis. The oxidative stress and inflammatory response microenvironment caused by PS-MPs may establish the ferroptosis NLRP3 inflammasome feedback pathway, which needs to be confirmed by subsequent targeted experiments.

In the overall regulatory network, Wnt/*β*-catenin and TGF-*β*/Smad pathways, as the core executor of fibrosis, receive multiple input signals from oxidative stress and immune inflammation. The TGF-*β* pathway is not only directly activated by oxidative stress (107), but also regulated by inflammatory factors, such as IL-6 (69). Similarly, the Wnt/*β*-catenin pathway is continuously activated in response to the stimulation of inflammation, which collaborates with the TGF-*β* pathway to promote EMT and fibroblast activation (108). The multi-dimensional interaction of immune-inflammation-fibrosis regulatory network makes the initial pulmonary inflammation and immune disorder induced by PS-MPs exposure, and may ultimately progress to pulmonary fibrosis-like pathology under sustained exposure.

### 5.4 Senescence

In the proposed pathological network linking polystyrene exposure to pulmonary fibrosis, oxidative stress, ferroptosis and inflammatory response constitute the three core modules of acute injury, while alveolar epithelial cell senescence is the fourth type of injury. However, this theoretical framework, with acute injury and classical inflammatory response as the core, has inherent limitations in explaining chronic and progressive pulmonary remodeling, such as pulmonary fibrosis, caused by long-term low-dose environmental exposure. For example, a combined exposure study for up to 16 weeks found that exposure to environmentally relevant doses of PS-NPs alone did not directly induce significant pulmonary fibrosis, but only exhibited severe damage when synergistically combined with pollutants such as benzo (a) pyrene (99). This suggests that in addition to acute toxicity, a more insidious and ongoing pathological mechanism must be driving the progression of chronic lung disease. Recent studies have revealed that cellular senescence may be the key link of this missing link. Cellular senescence is a state of irreversible cell proliferation arrest triggered by a variety of stressors such as DNA damage, telomere shortening, and oxidative stress. Although senescent cells stop dividing, they still maintain metabolic activity and have a profound impact on the surrounding microenvironment by secreting a large number of proinflammatory cytokines, chemokines, and matrix remodeling proteases, namely the senesence-associated secretory phenotype (SASP) (109). Under the PS-MPs exposure scenario, several studies have confirmed the involvement of cellular senescence from phenomena to mechanisms. For example, in a 35-day inhalation exposure experiment in Sprague-Dawley rats and in an *in vitro* experiment in the mouse alveolar epithelial cell line MLE12, PS-MPs treatment was observed to result in an increase in senescent *β*-galactosidase staining positive cells in lung tissue. The expression of senescence markers p21, p16 and p27 proteins was up-regulated, accompanied by increased secretion of SASP factors IL-6 and IL-8 (110). Another study also confirmed that all four typical microplastics could induce cellular senescence in human lung epithelial cells A549 and BEAS-2B, and antioxidant treatment could significantly reverse this phenotype (111). Therefore, shifting the research perspective from the simple axis of oxidative stress-acute inflammation-programmed death to oxidative stress-cell senescence provides a new perspective to understand how PS-MPs lead to persistent loss of function and abnormal remodeling of lung tissue.

Oxidative stress is the core bridge connecting PS-MPs exposure and cellular senescence. This continuous oxidative stress constitutes a common upstream signal that induces senescence markers such as mitochondrial dysfunction, telomere shortening, and DNA damage response (DDR) (9). In lung tissue, this accelerated cellular senescence, driven by oxidative stress, has important pathophysiological implications. Aging alveolar epithelial cells not only lose their normal repair and regeneration ability, but also secrete SASP factors through paracrine effect, which can activate the transformation of adjacent fibroblasts into myofibroblasts, promote collagen deposition, and directly promote the process of pulmonary fibrosis (69). At the same time, SASP mediated chronic low-grade inflammation can also remodel the immune microenvironment and aggravate the destruction and remodeling of the airway, which is highly consistent with the pathological characteristics of COPD and emphysema (112). At the molecular level, reactive oxygen species produced by oxidative stress can directly attack the telomere structure and induce telomere dysfunction, which in turn activates a complex DDR signaling network with ATM, ATR, p53/p21 and p16 as the core (113, 114). When the degree of DNA damage exceeds the repair capacity of the DDR, the accumulation of unrepaired damage triggers the cell to enter a state of irreversible proliferation arrest, that is, cellular senescence. Significant upregulation of cyclin-dependent kinase inhibitors such as p21 and p16, which are classical markers of cellular senescence, can be observed in alveolar epithelial cells exposed to PS-MPs (110). In addition, oxidative stress can induce calcium overload, lead to the accumulation of mitochondrial reactive oxygen species, and then activate the NF-κB signaling pathway. This process can also promote the expression of p21 and p16, and promote the process of cell senescence (115). Recent studies have also revealed new mechanisms of oxidative stress-mediated telomere damage. For example, mitochondrial ROS produced by oxidative stress can mediate the ubiquitination and degradation of telomeric repeat binding factor 2 (TRF2), resulting in loss of telomere protection and DNA damage. This process is regulated by m6A methylation modification, and ultimately drives the aging of alveolar epithelial cells and the progression of pulmonary fibrosis. ALKBH5 mediated FBXW7 m6A modification pathway is the key node to regulate the above cascade in 1-nitropyrene induced lung injury model. Although PS-MPs were not the exposed pollutants in this study, the molecular axis of mitochondrial ROS-mediated TRF2 degradation, telomere damage and cell senescence is universal, which supports the potential of sustained oxidative stress induced by PS-MPs to activate this aging pathway (116). In addition, increased ROS levels mediated cell senescence was also observed in A549 and BEAS-2B cells exposed to PVC microplastics (111). Therefore, oxidative stress-driven telomere damage combined with sustained DDR activation constitutes the molecular engine of PS-MPs induced alveolar epithelial cell senescence.

The SASP plays a key role in remodeling the lung microenvironment. SASP factors secreted by senescent lung epithelial cells not only consolidate their own aging state through autocrine way, but also act on neighboring cells, especially fibroblasts, through paracrine effects, thereby driving the process of pulmonary fibrosis. In IPF, the aging accumulation of alveolar epithelial cells disrupts the normal tissue repair program, and its release of pro-inflammatory mediators constitutes SASP and directly leads to the continuous activation of fibroblasts. The core mechanism of this process is that growth factors such as TGF-*β* secreted by senescent cells activate downstream signals through SMAD dependent and independent pathways, induce transdifferentiation of fibroblasts into myofibroblasts, produce abnormal ECM deposition, and eventually lead to tissue remodeling. In the PS-MPs exposure model, varying degrees of lung injury and fibrosis were observed in mice after microplastic inhalation, accompanied by significant increases in the expression of fibroblast activation markers such as α-SMA, Vimentin and Col1a, which further confirmed SASP as a paracrine hub to signal epithelial senescence to interstitial fibroblasts (15). Furthermore, In a model of bleomycin-induced pulmonary fibrosis, salvianolic acid B (SAB) treatment reversed the trend of SASP factor release in alveolar macrophages and alveolar type II (AT2) epithelial cells, leading to a significant reduction in lung fibroblast activation, underestimating the key SASP paracrine driver of fibroblast activation (117).

Another important remodeling effect of SASP on the lung microenvironment is reflected in the profound reprogramming of immune cell function. Studies have shown that the cGAS/STING signaling pathway is activated in PS-MPs-induced pulmonary fibrosis-like pathology in preclinical models, which is a key node for the innate immune system to sense cytoplasmic DNA and start the inflammatory response (15). Its continuous activation promotes the immune microenvironment from the early defensive response dominated by inflammatory cell infiltration to the profibrotic state characterized by immunosuppression and matrix remodeling (118). Macrophages play a central role in this transition. In the study of nanotubes induced pulmonary fibrosis, alveolar macrophages (AMs) undergo senescence accompanied by increased profibrotic SASP secretion, and the down-regulation of Sirt1 expression is a key factor promoting this transition (119). Senescent macrophages produce a large number of SASP factors, which not only aggravate the chronic inflammation of tissues, but also directly promote the proliferation of fibroblasts and ECM deposition, thus dragging the focus of the immune response from inflammation resolution to fibrosis (120). In conclusion, SASP accelerates the pathological process of chronic lung disease and pulmonary fibrosis by continuously activating immune cells and releasing pro-inflammatory and pro-fibrotic factors.

In the mechanism network of PS-MPs mediating pulmonary fibrosis, although studies on aging are few, it has unique pathological significance. First, aging explains how transient particle exposure induces a persistent fibrotic phenotype (81). This is mainly because of the stable cell cycle arrest and long-term survival characteristics of senescent cells, which enable them to continue to release SASP and maintain inflammatory and fibrotic signals after the termination of the initial exposure, which builds a mechanistic bridge between the high-dose acute exposure model in the laboratory and the low-dose chronic exposure in human life (111, 121). Secondly, Wnt/*β*-catenin signaling pathway and TGF-*β*/Smad signaling pathway play pivotal regulatory roles in the process of lung epithelial cell aging and fibrosis. In the aging mouse model, drugs can inhibit Wnt/*β*-catenin signaling pathway and reduce *β*-catenin into the nucleus, thereby alleviating bleomycin-induced pulmonary fibrosis and reducing the expression of aging markers (122). TGF-*β* itself is an important component of SASP and induces fibroblast activation. It has been shown that the profibrotic effects of TGF-*β* can be synergistically achieved through the Wnt/*β*-catenin pathway (108). Finally, targeted elimination of senescent cells provides new directions for intervention strategies. Du has demonstrated that attenuating epithelial senescence by inhibiting the PI3K/AKT/mTOR pathway with 5-methoxyindole-3-carboxaldehyde (5-MC) significantly alleviated fibrosis (123). Senolytic strategies—a group of drugs or strategies that can selectively induce the apoptosis of senescent cells. senolytic reduces inflammation and fibrosis by eliminating these senescent cells—aimed at clearing senescent cells have shown promising therapeutic potential in IPF models, suggesting that targeting cellular senescence may represent an important interventional entry point for PS-MPs associated pulmonary fibrosis.

### 5.5 Wnt/*β*-catenin and TGF-*β*1/Smad signal way

TGF-*β*/Smad pathway and Wnt/*β*-catenin pathway together constitute the core of fibrosis regulation. TGF-*β*/Smad signaling pathway is the key to regulate the synthesis and deposition of extracellular matrix, which plays an irreplaceable role in pulmonary fibrosis-like changes induced by PS-MPs in experimental models. TGF-*β*, as the main regulator of fibrosis progression, drives fibrosis progression by promoting the synthesis and deposition of ECM and inhibiting its degradation. Its signal transduction is regulated by classical Smad dependent and non-classical MAP kinase dependent pathways (108). PS-MPs exposure can directly activate this signal axis. For instance, airway instillation of polystyrene particles can induce the activation of the TGF-*β*1/Smad pathway in mice and secondary fibrosis lesions (16). At the cellular level, PS-NPs can induce EMT in human alveolar epithelial A549 cells, and the mechanism is related to the increase of ROS and the activation of NADPH oxidase 4 (NOX4) (74). In addition, PS-MPs can also initiate noncanonical TGF-*β* signaling branches, so that the profibrotic effect is not limited to the Smad-dependent transcriptional regulation mode (124). The central role of TGF-*β*1 led transcriptional programs involving miRNA regulatory effects that amplify the fibrotic phenotype has been well documented in the pathogenesis of pulmonary fibrosis (125). The profibrotic effect of TGF-*β*/Smad pathway does not exist in isolation, and it has a broad synergistic effect with Wnt/*β*-catenin pathway. In the process of fibrosis, TGF-*β*1 can upregulate ACP5 through the Smad3 pathway, which enhances its nuclear signal by stabilizing β-catenin protein, and jointly promotes the differentiation and proliferation of fibroblasts (126).

Wnt/*β*-catenin signaling pathway also plays a key profibrotic role in the process of pulmonary fibrosis-like pathology induced by PS-MPs. Repeated airway instillation of PS-MPs in mice can induce pulmonary fibrosis, which is accompanied by activation of Wnt/*β*-catenin pathway, increased oxidative stress and collagen accumulation (14). It can cooperate with TGF-*β*1 to continuously activate fibroblasts and hinder the repair of alveolar damage. For instance, inhibition of the Wnt/*β*-catenin pathway in human lung fibroblasts reduced the expression of TGF-*β*1-stimulated profibrotic markers, and Calcaratarin D, a natural compound, reduced bleomycin-induced pulmonary fibrosis in mice by inhibiting this pathway (127). The observation that TGF-β1 can upregulate β-catenin has been reported in other fibrotic disease models (128, 129), where it is established as a general feature of fibrogenic signaling rather than a PS-MPs-specific finding. These studies therefore provide mechanistic background for how TGF-β and Wnt pathways may interact, but they do not prove that this specific regulatory axis operates in PS-MPs-induced lung injury. Correspondingly, *β*-catenin promotes EMT and fibrosis process. Through mutual promotion and joint profibrotic mechanism, the two pathways form a bidirectional regulatory coupling relationship, making the TGF-*β*1/Smad-Wnt/*β*-catenin signaling complex a key signal integration hub. This integration hub ultimately initiates a stable expression that significantly upregulates profibrotic genes such as α-SMA, collagen, connective tissue growth factor, and fibronectin. Many factors, including oxidative stress, ferroptosis, mediators released by the inflammasome (IL-6, IL-1*β*, etc.) and aging-related secretory phenotype factors, can act and amplify the signal strength of this hub, and finally initiate the downstream terminal fibrosis process. Therefore, this review defines this dual-pathway signaling hub as the core mechanism fulcrum connecting microplastic-induced cellular stress with tissue level fibrosis and remodeling, and it is also the most potential node for targeted intervention.

This molecular logic of multi-module convergence has key interpretive value and is also the core theoretical framework constructed in this review. Each of the upstream damage modules described above can provide activation factor to the downstream signaling hub. Oxidative stress can initiate inflammation, ferroptosis and cellular senescence, which are also exacerbated by TGF-*β*/Smad pathway, forming a vicious cycle (130). Its oxidation products can promote TGF-*β* expression and enhance downstream signaling (95). IL-1*β* and IL-6 produced by inflammation can upregulate the expression of TGF-*β*1 and enhance the sensitivity of fibroblasts to TGF-*β*1 (121). Ferroptosis mediated epithelial cell death not only depletes the reserve of alveolar regeneration, but also releases lipid peroxide that changes the microenvironment (95). Senescent alveolar epithelial cells can secrete a large amount of TGF-*β* through SASP and change the lung microenvironment, further activating fibrosis signals (131). Although each of the upstream modules has been shown to be active after polystyrene exposure, the connectivity of these modules to the hub has not been tested. This merely proves that a pathway is activated in the system does not determine its position in the causal hierarchy.

### 5.6 EMT and ECM remodeling

The direct effects of signaling hub activation include EMT and ECM remodeling. When EMT occurs, alveolar epithelial cells lose apical-basal polarity and down-regulate epithelial markers such as E-cadherin. At the same time, mesenchymal markers such as α-SMA, vimentin, and N-cadherin were obtained and transformed into myofibroblast-like phenotype with the ability to migrate and synthesize matrix (74). PS-MPs and polyethylene micro/nanoplastics can induce EMT in bronchial and alveolar epithelial cells, accompanied by characteristic bidirectional changes of downregulation of epithelial markers and upregulation of mesenchymal markers (16, 132). In an animal model, inhalation of PS-MPs dose-dependently increased the expression of α-SMA, Vimentin, and Col1a in lung tissue, accompanied by a decrease in the alveolar epithelial marker SP-C, suggesting the involvement of EMT in fibrosis (14). Macrophage-derived paracrine signals play an important role in regulating EMT (69). It has been reported that protective factors such as NPY can alleviate EMT by inhibiting IL-1*β* release (133).

Activated myofibroblasts are the core effector cells in the process of fibrosis: these cells oversecrete collagen type I and III, fibronectin and other matrix components, and promote the contraction of the matrix, destroying the normal alveolar structure. Considering the clear relationship between EMT and TGF-*β*1/Smad as well as Wnt-*β*/catenin pathway, EMT and ECM remodeling can be regarded as the final effector stage when all upstream damage modules converge together.

In addition to phenotypic remodeling, the abnormally deposited ECM itself can actively drive fibrosis progression. The highly cross-linked and sclerotic ECM can activate the latent TGF-*β*1 through mechanical stress, and further promote the activation of myofibroblasts through mechanical transduction pathways. The matrix stiffness mediated feedforward loop is constructed, and the fibrotic tissue gradually enters a refractory hardening state (134). So, EMT and ECM remodeling may not be a simple downstream secondary effect, but actively participate in the positive regulation of fibrosis.

### 5.7 Epigenetic and ncRNA regulation

An additional regulatory layer stabilizes and finetunes the fibrotic program, epigenetic mechanisms and ncRNAs(ncRNAs). PS-MPs exposure can significantly change the expression profile of long non-coding RNA (lncRNA) and circular RNA (circRNA) in lung tissue, and functional studies have confirmed that such transcripts are involved in the regulation of pulmonary fibrosis response (13). Among them, the regulatory mechanism of circRNA-SMG6 is greatly clear. It mediates the dysfunction of pulmonary macrophages and epithelial cells induced by PS-MPs through the competitive endogenous RNA (miRNA sponge) mechanism, thereby affecting the metabolism of ECM and participating in the process of emphysema and fibrosis remodeling (135). Most ncRNAs exert their biological functions by targeted regulation of microrna (miRNA), and then relieve the inhibitory effect of miRNA on TGF-*β*1/Smad or Wnt pathway, and finally enhance the signaling activity of the profibrotic center. At the same time, TGF-*β*1 can also form its own feedforward loop by inducing specific miRNAs to continuously reinforce the fibrotic phenotype (125). Although epigenetic studies on PS-MPs associated pulmonary fibrosis are still limited, microplastic exposure may induce persistent epigenetic modifications, which can drive the continuous progression of fibrosis after the end of exposure (136). It also suggests that ncRNAs can be used as a potential biomarker and therapeutic target for lung injury in PS-MPs.

Despite significant mechanistic advantages, this regulatory module is still the least mature area of PS-MPs pulmonary fibrosis research. There is still a lack of sufficient causal verification, loss of function and gain of function experimental evidence in existing studies. Therefore, under the condition of real environmental exposure dose and duration, systematic epigenome and ncRNAs transcriptome analysis is the key research direction in this field.

## 6 Combined exposure and dose-response relation

### 6.1 Combined exposure, particle aging and host susceptibility

It is worth emphasizing that human inhalation exposures in real environments hardly involve raw, single PS-MPs under laboratory conditions. In the natural environment, it has been widely confirmed that microplastics can adsorb and enrich various co-pollutants through surface weathering, and act as carriers to co-transport co-pollutants into the cell interior (137). Particle aging is the core factor mediating this process: light and oxidative weathering can increase the specific surface area of microplastics, improve the surface roughness, generate a large number of oxygen-containing functional groups, and significantly enhance their adsorption capacity for heavy metals and organic pollutants (59). The toxicological consequence is that aged particles can deliver a composite chemical insult that amplifies injury at several of the mechanistic nodes described above—adsorbed polycyclic aromatic hydrocarbons, metals and persistent organic pollutants can independently generate ROS, activate inflammasomes and perturb signaling, potentially acting additively or synergistically with the particle itself (99, 138).

However, the interactions between microplastics and co-pollutants are not always shown as synergistic effects. According to the differences in the types of co-pollutants, physicochemical properties of particles and biological detection endpoints, the combined exposure can show different toxic effects such as additive, synergistic or antagonistic. For example, previous studies have confirmed that polystyrene particles can produce antagonistic effects with some co-exposed pollutants *in vivo*, and weaken their toxic effects by changing the bioavailability and distribution characteristics *in vivo* of co-exposed pollutants (139). In contrast, studies have explored this interaction using a 16-week-old mouse model exposed to environmentally relevant doses of polystyrene particles, benzo [a] pyrene, and their co-exposure. It was found that combined exposure could cause significant lung damage, increase oxidative stress and reduce antioxidant defense capacity in the lungs (99). This complex interaction suggests that traditional single-substance toxicological studies tend to systematically underestimate, or even occasionally overestimate, the health risks of real environmental exposures, highlighting the need for studies with mixed-exposure models. Host susceptibility adds another regulatory dimension to the pulmonary toxicity effects of microplastics. Patients with interstitial lung disease, obstructive pulmonary disease, the elderly, and individuals with genetic susceptibility to fibrosis are more sensitive to microplastic exposure. The repair ability and antioxidant reserve function of the lung epithelium have been damaged in such people. Microplastic exposure can further aggravates the progress of the original lung lesions (5). In conclusion, the synergistic effect of environmental combined exposure and host susceptibility may aggravate the damage intensity of the fibrotic mechanism network induced by microplastics.

In addition, the lung composite exposure system includes not only chemical pollutants adsorbed by microplastics, but also harmful inhaled factors widely existing in daily life, including combustion particles, cigarette smoke, endotoxin and respiratory pathogens. All kinds of exogenous stimuli can independently activate oxidative stress, inflammation and TGF-*β*1 fibrosis pathways, which highly coincide with the pathogenic targets of PS-MPs. This implies that multiple environmental damage signals can converge to the same core profibrotic hub, and PS-MPs are not the only causative factor, but an important auxiliary contributing factor in the cumulative exposure risk of pulmonary fibrosis. At present, the research framework that microplastics as environmental regulators and auxiliary causes of lung diseases rather than a single cause, has been increasingly recognized by the academic community. This theory has important guiding significance for population risk assessment and experimental design of susceptible hosts (140).

### 6.2 Dose-response relationships and the reference models for governing

Two questions must be separated when combined exposures are discussed: whether a dose–response relationship has been characterized, and on which null hypothesis model the observed anomalies are based. For PS-MPs alone, dose-dependent increases in α-SMA, vimentin and Col1a with a fall in SP-C have been reported after intratracheal administration in mice (14), and dose-and time-dependent TGF-*β*1 activation and fibrosis after intratracheal administration in rats (16). For administration, the available pulmonary data consist of a small number of fixed-ratio binary experiments. Polystyrene nanoplastics at environmentally relevant doses did not by themselves induce significant fibrosis over 16 weeks but did so when combined with benzo[a]pyrene (99). Polystyrene aggravated cadmium-induced mitochondrial damage in mouse lung (141). Nanoplastic–benzo[a]pyrene co-pollution aggravated pulmonary ferroptosis through endoplasmic-reticulum stress, with the particle acting as a carrier that increased cellular uptake of the PAH (142). Interactions are not uniformly synergistic. PS-MPs can act antagonistically *in vivo* by lowering the bioavailability of a co-exposed compound. No study of an inhaled PS-MPs mixture has yet reported a full dose–response surface, an isobolographic analysis—a classical mixture interaction analysis method, which can visually show the nature of the joint interaction of two substances—or a formal comparison against concentration-addition or independent-action predictions, and this remains the principal methodological gap in the field.

The two standard reference models should be stated explicitly. Concentration addition assumes similarly acting components and predicts the mixture effect from the sum of the toxic units of the component. Independent action assumes dissimilar modes of action and predicts the mixture effect from the product of the component survival probabilities. A particle carrying a sorbed organic chemical is a difficult case for both, because the components act by different mechanisms yet are not independent — the particle determines the delivered dose of the chemical. Quantitative appraisal of the wider mixture literature counsels caution before invoking synergy. Across binary pesticide, metal and antifouling mixtures, synergy defined as a two-fold departure from concentration addition occurred in 7%, 3% and 26% of cases respectively and rarely exceeded ten-fold (143). A systematic reappraisal of 1220 mixture experiments found that most reported synergies fell within a factor of two of additive predictions (144). Thus, additivity should be the default treatment for PS-MP mixtures, and deviations from this principle need to be tested experimentally rather than being directly assumed.

### 6.3 Shift in the dominant toxic module

The relative contribution of distinct toxic modules is unlikely to remain constant across the full dose range. Instead, we propose that two mechanistically distinct regimes operate, with a dose-dependent transition between them that is both explicit and testable. The first is the carrier mechanism. In this mechanism, the particle functions primarily as a delivery vehicle. Its contribution depends on the concentration gradient between the sorbed chemical and the receiving tissue, and it operates only while the chemical load on the particle exceeds that in the surrounding tissue. Model-supported reanalysis of vector effects in other compartments indicates that the quantitative importance of this carrier effect is often smaller than assumed once competing exposure routes are considered (145). Particle aging modulates this regime substantially. For instance, weathering increases specific surface area and generates oxygen-containing surface functionalities that raise sorption capacity, while surface chemistry governs desorption as well as adsorption — meaning that aged particles not only carry more chemical but also release it differently.

The second is the particle-intrinsic character. Toxicity scales with retained particle mass and surface area rather than with adsorbed chemical load. Once deposition outpaces macrophage-mediated clearance, the system enters a state characterized by frustrated phagocytosis, overload of clearance mechanisms, and chronic foreign-body macrophage activation — a regime well described for other poorly soluble, low-toxicity particles. Crucially, the transition between these two regimes is self-reinforcing. As retained PS-MPs accumulate, they themselves delay macrophage-mediated clearance, which in turn promotes further retention and accelerates the shift toward the particle-intrinsic character. We therefore predict a dose-dependent inversion of the dominant mechanism. At environmentally relevant retained burdens, the adsorbed chemical load is expected to dominate the response, with the particle acting mainly as a carrier. At the high burdens characteristic of bolus instillation studies, by contrast, the particle-intrinsic module is expected to dominate, with the chemical contribution becoming proportionally minor. The most direct experimental support for such an inversion comes from human A549 cells. At 20 μg mL^−1^, polystyrene nanoplastics reduced the joint toxicity of phthalate esters by lowering their freely dissolved concentration (a carrier-mediated antagonistic effect), whereas at 200 μg mL^−1^ the combined toxicity became dominated by the particle itself (146). The prediction is falsifiable. Carrier-dominated injury should be accompanied by markers of the carried chemical, such as aryl hydrocarbon receptor activation and CYP1A1 induction for PAHs or metallothionein induction for cadmium, whereas particle-dominated injury should track retained particle surface area, macrophage burden and NLRP3-dependent IL-1β release. An important corollary is that studies conducted exclusively at bolus doses may systematically underestimate the contribution of adsorbed chemicals at the exposures humans actually experience.

Because carrier-dominated and particle-intrinsic regimes preferentially amplify different downstream injury modules — the former tracking the toxicological signature of the carried chemical and the latter favoring NLRP3-driven foreign-body inflammation — this dose-dependent inversion has direct implications for the relative contribution of the four modules defined in **Figure 2**. Moreover, different adsorbed pollutants can exert their toxic effects through different modules. Inflammation, ferroptosis, or oxidative stress may all play a dominant role. It is worth emphasizing that oxidative stress may be a shared mechanism of toxicity for various particles.

## 7 Integration: a mechanistic network from inhalation to fibrosis

Synthesizing the foregoing modules yields a coherent, multi-scale mechanistic network (**Figure 2**). This pathogenic pathway starts from the core physicochemical characteristics of airborne PS-MPs, including particle size, surface charge, protein corona and pollutant loading, which together determine the deposition efficiency of particles in alveoli and the level of endocytosis and internalization of alveolar epithelial cells and macrophages (**Table 2**). The biorefractory particles that enter cells continuously accumulate in lysosomes and mitochondria, directly triggering the early oxidative stress burst. This oxidative signal is the core branch node that initiates multiple downstream parallel damage modules. On the one hand, it regulates the process of ferroautophagy by activating YY1/FTL and cGAS-STING pathways, and mediates iron-dependent epithelial ferroptosis. On the other hand, it induces endoplasmic reticulum stress and mitochondrial dysfunction, which provides the initiating signal for the assembly of NLRP3 inflammasome and the release of IL-1*β* and IL-6. At the same time, it pushes the surviving alveolar epithelial cells into a senescent state and secretes a large number of profibrotic SASP factors. These modules are not isolated but cross-talk extensively. Ferroptosis and pyroptosis release damage-associated molecular patterns that further activate innate immunity. cGAS–STING can bridge mitochondrial damage to both ferroptosis and inflammation, and the SASP sustains the inflammatory milieu.

**Table 2:** Influence of particle physicochemical heterogeneity on pulmonary cellular responses to polystyrene microplastics.

Property	Effect on deposition, uptake and toxicity	References
Size (smaller *→* greater effect)	Deeper alveolar deposition, higher surface-area-to-mass ratio, more efficient endocytosis, greater ROS and cytotoxicity; nanoscale fractions can translocate systemically	(27, 38, 80, 81)
Surface charge (cationic vs. an-ionic)	Charge-dependent uptake and trafficking; cationic (amine) particles generally internalized more and more membranolytic/lysosomotropic than anionic (carboxyl)	(30, 31)
Biomolecular corona	Protein/lipid adsorption reshapes effective surface, modulates uptake route and can dominate particle–cell interaction	(34, 35)
Additives, batch & supplier variability	producing large variability in measured toxicity	(34, 36)
Aging/weathering	Increased surface oxidation and roughness enhance co-pollutant adsorption and can alter intrinsic toxicity	(59, 137, 138)
Polymer type (PS vs. PP, PET, PE, tire wear)	Polymer identity modulates inflammatory potency and fibrogenic endpoints	(55, 62, 132)

Effects are generalized trends from the cited literature; direction and magnitude depend on cell type, dose and assay.

In the above injury network, the core event is that all kinds of upstream damage signals converge together in the TGF-*β* 1/Smad and Wnt/*β* - catenin coupling signaling hub. Cytokines and epithelial SASP factors released by inflammasome activation can directly promote TGF-*β*1 production. Oxidative stress can reduce the activation threshold of the two core pathways. The ferroptosis process is closely interregulated with the EMT program mediated by the hub. Once continuously activated, the signaling hub can simultaneously drive the EMT of alveolar epithelial cells and promote the differentiation of fibroblasts into myofibroblasts with matrix secretion and contraction functions. At the same time, epigenetic regulation and ncRNA programs, including circRNA-SMG6 and TGF-*β*1 regulated miRNA, further target the interstitial profibrotic transcriptional phenotype and allow the injury state to stabilize. The final effects of this pathway cascade are excessive deposition and cross-linking of collagen and other ECM, destruction of normal alveolar structure, progressive decline of lung function, and finally formation of typical pathological features of pulmonary fibrosis. Combined environmental exposure and host susceptibility were distributed at multiple upstream regulatory nodes, continuously amplifying the overall damage intensity.

This multi-dimensional pathogenic network has double core features, which are particularly critical for understanding the mechanism and intervention strategy of fibrosis associated with PS-MPs exposure. First, although various upstream damage pathways have different mechanisms of action, they all provide activation signals to the core hub of TGF-*β*1/Smad and Wnt/*β*-catenin to form a unified downstream profibrotic output. This feature explains why a consistent phenotype of pulmonary fibrosis has been observed in multiple independent studies that have used different particle types, exposure doses, and measures. Second, there are multiple self-enhancing loops in the network, and mitochondrial-derived ROS can further exacerbate oxidative stress and promote continuous accumulation of ROS. The damage-related molecular pattern released by ferroptosis and pyroptosis can continuously activate innate immune cells and amplify inflammatory damage. Senescent cells recruit and activate a large number of fibroblasts by secreting SASP. Sclerotic ECM mechanically activates latent TGF-*β*1. Once the damage crosses a critical threshold, the fibrotic lesions can progress independently from the persistent particle exposure. The understanding of these two points provides the core theoretical basis for the screening of subsequent intervention targets and the optimization of prevention and control strategies. There are three features of the available evidence that deserve to be highlighted that delineate the interpretive boundaries of the mechanistic framework. First, the evidence supporting each component of the framework is uneven. Each individual injury module is supported by direct data from PS-MPs lung experiments, but the convergence effects of upstream injury events and the coupling relationships within the core signaling hub are partly extrapolated from other relevant models. Second, the phenotypic consistency observed across independent studies - namely, repeated reports of oxidative stress, epithelial-cell death, inflammation, and collagen accumulation – has limited probative value in demonstrating the existence of a uniform cascade. The above series of pathological changes are the response of alveolar structure to various persistent injuries. Regardless of whether the lesion module is triggered via PS-MPs, similar pathological phenotypes may result. Third, no study has targeted an intervention to a single damage module within the same set of PS-MPs exposure systems and examined the subsequent effects of this operation on other damage modules as well as the downstream output of the hub. This experimental design is the bare minimum required to advance the descriptive network model to the level of formal causal inference.

The conceptual framework established in this review is based on three premises. To begin with, documented evidence of human exposure to microplastic particles found in human respiratory tissues from observational clinical studies confirms that humans do instill and retain microplastics. What’s more, experimental fibrotic response studies have shown that PS-MPs are able to trigger fibrosis-like pathological changes under laboratory conditions. Last, mechanistically, these experimental observations support mechanistic plausibility that microplastic exposure may contribute to fibrotic changes. However, it remains unclear whether humans will actually be affected. The available human data are all cross-sectional, and it is unclear whether microplastic exposure precedes fibrosis or whether decreased lung clearance in the disease state leads to the accumulation of microplastics. Longitudinal, dose-based epidemiological studies are helpful to further understand this issue. There is currently insufficient epidemiological evidence to confirm the existence of a relevant causal relationship in humans. Accordingly, this integrated pathogenic network serves as a hypothesis-generating mechanistic framework informing future toxicological and clinical investigations.

## 8 Therapeutic interventions and translational implications

The above multi-dimensional pathogenic network provides multiple reasonable intervention targets for PS-MPs associated pulmonary fibrosis. The most fundamental transformation strategy is primary prevention, which is to reduce the generation and human exposure level of airborne PS-MPs through material substitution, pollution source control and indoor air quality optimization. Primary prevention is more critical than pharmacologic intervention, because no drug can completely reverse persistent, biorefractory particle-mediated chronic lung injury. For established lung injury, the signal aggregation characteristics of this mechanistic network suggest that targeting shared profibrotic signaling hubs is an efficient intervention idea.

At present, the clinically approved anti-fibrotic drugs Nintedanib and Pirfenidone can inhibit the progression of fibrosis in a broad spectrum. Nintedanib blocks the signal transduction pathways of multiple profibrotic growth factors by inhibiting receptor tyrosine kinases (147). Pirfenidone can effectively inhibit TGF-*β*1-mediated activation of fibroblasts, and has anti-inflammatory and antioxidant activities (148). Their transferability to PS-MPs-associated fibrosis is unproven and cannot be assumed, for reasons that are structural rather than pharmacological. Neither agent has any described action on particle uptake, macrophage clearance capacity or polymer degradation, and polystyrene particles is not enzymatically degraded. The retained particle burden therefore continues to supply ROS, damage-associated molecular patterns and senescence-associated secretory factors to every upstream node of the network for as long as it remains in the lung, and this input is further sustained because retained particles themselves delay clearance. The medicine can constrain the output of that network but cannot reduce its input. The realistic expectation is therefore a reduction in the rate of progression rather than its termination — mirroring their clinical effect in idiopathic pulmonary fibrosis which is also what is observed clinically in idiopathic pulmonary fibrosis.

The closest experimental analogue is silicosis, in which the injurious agent is likewise retained and non-degradable. Pirfenidone and nintedanib each improved lung function and reduced fibrosis in mice with established silicosis, and the combination outperformed either monotherapy even at low doses (149). Inhaled nintedanib nanosuspensions have also been developed for the same model (150). The analogy has limits, since crystalline silica is directly cytotoxic and a potent inflammasome agonist, whereas polystyrene particles is comparatively inert, so the relative contribution of the injury modules and hence the expected drug response may differ. Moreover, no biomarker currently identifies microplastic-attributable fibrosis in an individual patient, so a trial population could not at present be defined. The decisive experiment is straightforward but has not been done. Administration of nintedanib or pirfenidone in a chronic, environmentally anchored PS-MPs inhalation model, with retained particle burden quantified alongside lung function and histological fibrosis.

PS-MPs exposure induces alveolar epithelial senescence and a senescence-associated secretory phenotype. Senescent cells are causally implicated in fibrosis in other models, and senolytic drugs reduce fibrotic markers in lung tissue from patients with idiopathic pulmonary fibrosis ex vivo. However, five practical problems limit the application of such drugs in microplastic-related pulmonary fibrosis. First, human pulmonary experience is limited to a first-in-human open-label pilot of intermittent dasatinib plus quercetin in 14 patients with IPF (151) and a subsequent randomized, placebo-controlled Phase I trial in 12 patients (152). The studies only verified the feasibility and tolerance of the drug, but did not focus on the anti-fibrosis effect, and could not provide effective clinical evidence. Second, BCL-2/BCL-xL inhibitors of the navitoclax class cause on-target thrombocytopenia, since platelet survival depends on BCL-xL. Third, senescence is heterogeneous and lacks a specific marker, so the selectivity of senolysis is defined operationally by the drug rather than by the target cell. Fourth, Transient cell senescence is involved in normal wound repair, and there is overlap between senescent alveolar cells and type II alveolar progenitor cells, the depletion of which can directly induce fibrosis. Non-specific elimination of senescent cells may damage the repair and regeneration of alveoli and aggravate lung injury. Fifth, Pulmonary retained particles will continue to induce cell senescence, resulting in the continuous regeneration of senescent cells, so long-term, intermittent administration, and possibly lifelong intervention are needed. No senolytic has been evaluated in any microplastic exposure model.

The PS-MPs-specific evidence here is associative rather than causal. Exposure alters lncRNA and circRNA expression profiles in lung tissue (13). CircRNA-SMG6 has been proposed to mediate macrophage and epithelial dysfunction through a competing-endogenous-RNA mechanism (135). And circRNA_kif26b has been linked to alveolar epithelial senescence via miR-346-3p (110). However, none of these studies establishes that the transcript is required for fibrosis *in vivo*.

The clinical precedent for RNA-directed antifibrotic therapy is sobering and belongs in a balanced perspective. MRX34, a liposomal miR-34a mimic, was terminated in phase 1 after immune-related serious adverse events (153). MRX34 was the first miRNA mimetic to enter clinical trials, and its failure triggered widespread concern about the immunogenicity and safety of miRNA therapy. Lademirsen, an anti-miR-21 oligonucleotide with a strong preclinical antifibrotic rationale, did not slow kidney function decline in a randomized controlled trial in Alport syndrome (154). Remlarsen, a miR-29 mimic, has shown target engagement only in intradermal proof-of-concept studies in skin (155). Beyond efficacy, the generic obstacles to RNA therapeutics, such as delivery, stability, off-target activity, immunogenicity and the difficulty of achieving cell-type specificity, remain incompletely solved (156).

In addition to the hub targeting strategy, targeted intervention for upstream damage nodes is also feasible. Given the central driver of oxidative stress in PS-MPs lung injury, targeted antioxidant strategies, including enhancing mitochondrial antioxidant reserve and activating Nrf2/HO-1 signaling axis, can effectively block the upstream early damage-amplifying loop, and the therapeutic value of Nrf2 pathway in pulmonary fibrosis has been widely confirmed (72, 73). As the key driving link of PS-MPs mediated alveolar epithelial damage, ferroptosis inhibition strategies may become the intervention direction with strong mechanism specificity. Ferroptosis has been recognized as a potential intervention target for pulmonary fibrosis (86, 89). Similarly, inhibition of cGAS-STING pathway or NLRP3 inflammasome activation can effectively block the activation effect of core hub mediated by inflammation (91, 102). In addition, circRNA-SMG6 and other ncRNAs can act on the early regulation of injury and are promising new intervention targets and noninvasive biomarkers (13, 135). The existing review studies generally pointed out that PS-MPs can be used as an important environmental regulator to participate in the progression of lung diseases. The clinical prevention and control of PS-MPs should be combined with exposure prevention and mechanism targeted drug intervention, and focus on the susceptible population of lung diseases and patients with basic lung diseases (49, 140). Based on the hierarchical architecture of the pathogenic network, the existing intervention strategies can be systematically divided into three types of upstream, middle and downstream targeting models, and each strategy has its own advantages and limitations. The upstream proximal intervention strategy, represented by antioxidants, Nrf2 activators and mitochondria-targeted free radical scavengers, can block the pathogenic signal transduction at the early stage of the injury cascade and prevent the progress of damage from the source. However, such strategies have obvious shortcomings: continuous residual microplastic particles can continuously regenerate oxidative stress signals, and broad-spectrum antioxidant regimens still lack sufficient efficacy support in the clinical application of pulmonary fibrosis. The middle-level module targeting strategy, which mainly consists of ferroptosis inhibitors, iron chelating agents, cGAS-STING antagonists and NLRP3 inhibitors, has stronger mechanism specificity and is supported by a large number of PS-MPs specific experimental data, The main limitation of such strategies is the functional redundancy of the pathogenic network: blocking a single death or inflammation module can be compensated by other upstream damage pathways, which continuously input activation signals to the core profibrotic hubs, resulting in limited intervention effects. The downstream hub and effector targeting strategy is represented by the clinically approved Nintedanib and Pirfenidone (147, 148), which directly acts on the signal aggregation hub and the terminal matrix remodeling process, which is not easy to be compensated by a single upstream module, and the intervention is more stable. However, these drugs cannot remove the initial particle stimulation, and can only delay rather than terminate the progression of fibrosis, which is consistent with the bottleneck of clinical treatment of IPF. Considering the advantages and disadvantages of various strategies, the combined intervention program of exposure prevention and control combined with core hub anti-fibrosis drugs and specific targeted drugs is significantly superior to the traditional single-target monotherapy mode for persistent PS-MPs microparticle lung injury. At the same time, accurate stratified diagnosis and treatment and efficacy monitoring rely on specific biomarker systems, including lung tissue particle load level, oxidative stress and ferroptosis related indexes, circulating ncRNAs, which can provide support for patient stratification, efficacy evaluation and prognosis monitoring.

## 9 Limitations and future directions

Despite the rapid progress of relevant research, the existing evidence system still has many limitations. At the same time, there is a significant difference between the original commercial particles used in the experiment and the environmentally aged microplastics. In addition, there are technical bottlenecks in the accurate quantitative detection of PS-MPs in tissue samples, and the inherent defects of various *in vitro* culture and animal tracheal instillation models limit the accuracy of data in the interdisciplinary field of environmental medicine. The relevant issues have been addressed above.

The differences in toxicological effects caused by long-term low-dose exposure and acute high-dose exposure of PS-MPs need to be carefully discussed. Acute high-dose exposure model, usually refers to the single or multiple administration of high doses of PS-MPs in a short period of time, can rapidly induce severe oxidative stress, mitochondrial dysfunction and apoptosis, leading to acute lung injury and early fibrosis-like pathology (80, 157). Aged fragmented PS-NPs (about 100 μg/animal) could increase the number of neutrophils by 3.9-fold after a single exposure (78). This type of acute model is helpful for the rapid screening of key toxic pathways and intervention targets, but the injury pattern triggered by this model may be biased toward acute inflammation-repair response, which is different from the nature of chronic progressive fibrotic diseases (81). In contrast, the long-term low-dose exposure model focuses on simulating the real environmental exposure, and what is observed is a chronic and progressive pathological process. For example, 28 to 35 days of inhalation exposure can lead to oxidative dysregulation and cellular senescence in the lungs (80, 110). Notably, the harmful effects of PS-NPs, such as lung barrier disruption, inflammation, and fibrosis, persisted even after the cessation of exposure, suggesting that the injury may be irreversible (81). This exposure pattern reveals mechanisms that are more consistent with the development of clinical fibrosis, such as chronic oxidative stress accumulation, depletion of antioxidant defense systems, and cellular senescence.

In addition to this, several key issues remain that have not been fully explored. First, the human-level evidence consists almost exclusively of cross-sectional association studies. Although microplastics have been detected in human lung tissue and blood, no longitudinal epidemiological study with dose stratification has confirmed the causal association between environmental PS-MPs exposure and new-onset pulmonary fibrosis. Therefore, it is of great research value to construct prospective cohorts that can quantify the respiratory particle burden. Second, the development of mechanism research is uneven. Oxidative stress, ferroptosis, and TGF-*β*1/Wnt pathway are relatively well studied, while epigenetic and ncRNA regulation, the role of lung microbiome, gender differences, and the effect of nanoplastics (different from microparticles) remain to be further explored. Third, the research on the relationship between combined exposure and host susceptibility is still in its infancy. There is an urgent need to carry out toxicological research on mixed pollutants and model experiments on basic lung diseases to support real environmental risk assessment. A further critical limitation concerns the absence of adjusted epidemiological analyses. Existing cross-sectional studies that associate microplastic detection in bronchoalveolar lavage fluid or lung tissue with fibrotic phenotypes rarely employ multivariable regression to account for established confounders — including smoking history, occupational dust and chemical exposure, and pre-existing chronic lung disease — each of which can independently influence both particle retention and fibrotic progression. Without such adjustment, reported associations cannot be reliably attributed to microplastic exposure itself, and the possibility that they are driven by confounding or reverse causation remains open. Establishing whether inhaled microplastics are causally linked to pulmonary fibrosis in humans will require prospective cohort studies with comprehensive covariate adjustment, validated quantitative exposure biomarkers, and dose-resolved longitudinal follow-up. In addition to the above problems, the whole field also faces the common problem of lack of standardization, which hinders the integration of different research results. The particle size distribution, surface chemistry, additives and endotoxin contamination levels of PS-MPs from different manufacturers, batches and laboratories were uneven. Various studies use different dose units such as mass, particle number and concentration, and lack of standardized treatment of deposition surface area, which makes it difficult to compare and even contradictory results of different studies (34, 36). The first is to confirm the particle characterization information of environmentally representative materials (notably particle size, Zeta potential, biomolecular corona, and endotoxin level), the second is to uniformly use the deposition surface area as the dose index, and finally, the toxicity analysis is performed according to the standard reporting guidelines established by toxicology.

The last key direction is to get rid of the single PS-MPs exposure model and get closer to the complex pollutant system and susceptible host in the real population exposure scenario. The key to the transformation from mechanism research to risk assessment is to carry out PS-MPs combined with the coexistence of inhaled pollutants, to replace the original particles with aging particles, and to construct the underlying lung disease model.

## 10 Conclusion

In the scenario of human respiratory exposure, inhaled PS-MPs have changed from a potential hazard to a proven exposure component, and experimental data have built a coherent, hypothesis-generating mechanistic framework that connects PS-MPs exposure with the risk of pulmonary fibrosis. The physicochemical properties of such biopersistent particles determine their level of deposition in the alveoli, as well as their efficiency of internalization by epithelial cells. Particle internalization initiates an early burst of oxidative stress, which further activates multiple cross-talk injury modules, such as ferroptosis via cGAS-STING, YY1/FTL and ferroautophagy pathways, NLRP3 mediated inflammation, and alveolar epithelial cell senescence. These damage modules eventually converge to the TGF-*β*1/Smad-Wnt/*β*-catenin coupling signaling hub, driving EMT, myofibroblast activation and ECM remodeling. On this basis, epigenetic and ncRNA regulatory pathways maintain the stability of fibrosis phenotype. Combined exposure can amplify the damage effect at multiple regulatory nodes. In the future, it is necessary to construct chronic inhalation models and lung organoids that are more suitable for human exposure characteristics, and to reproduce the three-dimensional structure of air-liquid interface, mechanical stretch and multicellular coexistence microenvironment *in vitro*, so as to achieve reliable toxicity assessment and intervention program verification. Single-cell multi-omics and spatial transcriptome technology are helpful to analyze the heterogeneous role and dynamic evolution of different cell subsets in the process of fibrosis. In addition, the toxic mechanism of the combined exposure of microplastics and benzo (a) pyrene, organophosphorus esters and other environmental pollutants should be further explored, and stable biomarkers related to oxidative stress, cell death and cell senescence should be screened based on prospective cohorts to provide support for environmental exposure risk assessment and early warning. From the breakthrough of basic mechanism research to the clinical application of prevention and control, it is necessary to integrate the multidisciplinary forces of toxicology, materials science, respiratory medicine and public health to promote the research and development of targeted intervention strategies based on pathogenic mechanisms. At the same time, scientific research evidence should be translated into environmental governance policies and long-term health management plans to actively respond to the increasingly severe respiratory health threat caused by microplastic pollution.

## References

[1] Geyer R Jambeck JR Law KL . Production, use, and fate of all plastics ever made. Sci Adv. (2017) 3:e1700782. doi: 10.1126/sciadv.1700782 28776036 PMC5517107

[2] Munyaneza J Jia Q Qaraah FA Hossain MF Wu C Zhen H . A review of atmospheric microplastics pollution: In-depth sighting of sources, analytical methods, physiognomies, transport and risks. Sci Total Environ. (2022) 822:153339. doi: 10.1016/j.scitotenv.2022.153339 35077799

[3] Cox KD Covernton GA Davies HL Dower JF Juanes F Dudas SE . Human consumption of microplastics. Environ Sci Technol. (2019) 53:7068–74. doi: 10.1021/acs.est.9b01517 31184127

[4] Cao G Cai Z . Getting health hazards of inhaled nano/microplastics into focus: expectations and challenges. Environ Sci Technol. (2023) 57:3461–3. doi: 10.1021/acs.est.3c00029 36812144 PMC9996812

[5] Vasse GF Melgert BN . Microplastic and plastic pollution: Impact on respiratory disease and health. Eur Respir Rev. (2024) 33. doi: 10.1183/16000617.0226-2023 39009408 PMC11262622

[6] Özgen Alpaydin A Uçan ES Köktürk M Atamanalp M Kalyoncu Ç Yiğit S . Microplastics, as a risk factor in the development of interstitial lung disease- a preliminary study. Environ pollut. (2024) 363:125054. doi: 10.1016/j.envpol.2024.125054 39368622

[7] Momeni MK Taghipour H Ghayebzadeh M Mohammadi M Keikhaee R . Isolation and characterization of microplastics from the human respiratory system: Sputum, broncho-alveolar lavage fluid, and pleural fluid simultaneously. Environ pollut. (2025) 365:125389. doi: 10.1016/j.envpol.2024.125389 39608742

[8] Banerjee A Billey LO Shelver WL . Uptake and toxicity of polystyrene micro/nanoplastics in gastric cells: Effects of particle size and surface functionalization. PloS One. (2021) 16:e0260803. doi: 10.1371/journal.pone.0260803 34971556 PMC8719689

[9] Mahmud F Sarker DB Jocelyn JA Sang QA . Molecular and cellular effects of microplastics and nanoplastics: Focus on inflammation and senescence. Cells. (2024) 13. doi: 10.3390/cells13211788 39513895 PMC11545702

[10] Olson AL Gifford AH Inase N Fernández Pérez ER Suda T . The epidemiology of idiopathic pulmonary fibrosis and interstitial lung diseases at risk of a progressive-fibrosing phenotype. Eur Respir Rev. (2018) 27. doi: 10.1183/16000617.0077-2018 30578336 PMC9489016

[11] Mazurek JM Syamlal G Weissman DN . Idiopathic pulmonary fibrosis mortality by industry and occupation - United States 2020-2022. MMWR Morb Mortal Wkly Rep. (2025) 74:109–15. doi: 10.15585/mmwr.mm7407a1 40048397 PMC11956160

[12] Kern DG Crausman RS Durand KT Nayer A Kuhn C . Flock worker's lung: chronic interstitial lung disease in the nylon flocking industry. Ann Intern Med. (1998) 129:261–72. doi: 10.7326/0003-4819-129-4-199808150-00001 9729178

[13] Fan Z Xiao T Luo H Chen D Lu K Shi W . A study on the roles of long non-coding RNA and circular RNA in the pulmonary injuries induced by polystyrene microplastics. Environ Int. (2022) 163:107223. doi: 10.1016/j.envint.2022.107223 35390562

[14] Li X Zhang T Lv W Wang H Chen H Xu Q . Intratracheal administration of polystyrene microplastics induces pulmonary fibrosis by activating oxidative stress and Wnt/β-catenin signaling pathway in mice. Ecotoxicol Environ Saf. (2022) 232:113238. doi: 10.1016/j.ecoenv.2022.113238 35121255

[15] Zhang J Du J Liu D Zhuo J Chu L Li Y . Polystyrene microplastics induce pulmonary fibrosis by promoting alveolar epithelial cell ferroptosis through cGAS/STING signaling. Ecotoxicol Environ Saf. (2024) 277:116357. doi: 10.1016/j.ecoenv.2024.116357 38677073

[16] Xia S Yuan C Long W Wu Z Li X Wang N . Intratracheal administration of polystyrene micro(nano)plastics with a mixed particle size promote pulmonary fibrosis in rats by activating TGF-β1 signaling and destabilizing mitochondrial dynamics and mitophagy in a dose- and time-dependent manner. Toxics. (2025) 13. doi: 10.3390/toxics13060487 40559960 PMC12197594

[17] Bu W Jin Y Zhou Y Wang F Zheng D Liu R . Polystyrene nanoplastics-induced lung epithelial cells ferroptosis promotes pulmonary fibrosis via YY1/FTL axis. Mater Today Bio. (2026) 36:102738. doi: 10.1016/j.mtbio.2025.102738 41560817 PMC12813319

[18] O'Brien S Rauert C Ribeiro F Okoffo ED Burrows SD O'Brien JW . There's something in the air: A review of sources, prevalence and behaviour of microplastics in the atmosphere. Sci Total Environ. (2023) 874:162193. doi: 10.1016/j.scitotenv.2023.162193 36828069

[19] Zhang Y Kang S Allen S Allen D Gao T Sillanpää M . Atmospheric microplastics: A review on current status and perspectives. Earth Sci Rev. (2020) 203:103118. doi: 10.1016/j.earscirev.2020.10311842574925

[20] Zhang M Xu L . Transport of micro- and nanoplastics in the environment: Trojan-horse effect for organic contaminants. Crit Rev Environ Sci Technol. (2022) 52:810–46. doi: 10.1080/10643389.2020.184553137339054

[21] Kim S Fesenmeier DJ Park S Torregrosa-Allen SE Elzey BD Won YY . Pulmonary pharmacokinetics of polymer lung surfactants following pharyngeal administration in mice. Biomacromolecules. (2022) 23:2471–84. doi: 10.1021/acs.biomac.2c00221 35580262 PMC12001817

[22] Wu Z Guo R Zhou Y Bi X Lin LL Ye J . Predominance of the noncell-mediated migration mechanism for large nanoparticles into sentinel lymph nodes. ACS Nano. (2026) 20:18428–43. doi: 10.1021/acsnano.6c05667 42289981

[23] Kamalasekaran K Sundramoorthy AK . A review on microplastics: sources, environmental fate, degradation pathways, and analytical identification methods. RSC Adv. (2026) 16:13851–74. doi: 10.1039/d5ra09062h 41837161 PMC12981036

[24] Mostovenko E Canal CG Cho M Sharma K Erdely A Campen MJ . Indirect mediators of systemic health outcomes following nanoparticle inhalation exposure. Pharmacol Ther. (2022) 235:108120. doi: 10.1016/j.pharmthera.2022.108120 35085604 PMC9189040

[25] Dong CD Chen CW Chen YC Chen HH Lee JS Lin CH . Polystyrene microplastic particles: *in vitro* pulmonary toxicity assessment. J Hazard Mater. (2020) 385:121575. doi: 10.1016/j.jhazmat.2019.121575 31727530

[26] Goodman KE Hare JT Khamis ZI Hua T Sang QA . Exposure of human lung cells to polystyrene microplastics significantly retards cell proliferation and triggers morphological changes. Chem Res Toxicol. (2021) 34:1069–81. doi: 10.1021/acs.chemrestox.0c00486 33720697

[27] Shi X Wang X Huang R Tang C Hu C Ning P . Cytotoxicity and genotoxicity of polystyrene micro- and nanoplastics with different size and surface modification in A549 cells. Int J Nanomedicine. (2022) 17:4509–23. doi: 10.2147/ijn.S381776 36186531 PMC9519127

[28] Chen L Liu Y Li H Lin S Wang X Fang J . Size-dependent pulmonary toxicity and whole-body distribution of inhaled micro/nanoplastic particles in male mice from chronic exposure. Environ Sci Technol. (2025) 59:6993–7003. doi: 10.1021/acs.est.4c14232 40181497

[29] Shanmugiah J Kim S Jeong H Kong J Lee SS Kang DK . Size-dependent pulmonary toxicity of inhaled micro- and nano-polystyrene and initial identification of microplastics in human lung cancer tissue. J Hazard Mater. (2026) 508:141897. doi: 10.1016/j.jhazmat.2026.141897 41916175

[30] Jeon S Clavadetscher J Lee DK Chankeshwara SV Bradley M Cho WS . Surface charge-dependent cellular uptake of polystyrene nanoparticles. Nanomaterials (Basel). (2018) 8. doi: 10.3390/nano8121028 30544753 PMC6316338

[31] Liu YY Liu J Wu H Zhang Q Tang XR Li D . Endocytosis, distribution, and exocytosis of polystyrene nanoparticles in human lung cells. Nanomaterials (Basel). (2022) 13. doi: 10.3390/nano13010084 36615994 PMC9824409

[32] Chen J Xu Z Liu Y Mei A Wang X Shi Q . Cellular absorption of polystyrene nanoplastics with different surface functionalization and the toxicity to RAW264.7 macrophage cells. Ecotoxicol Environ Saf. (2023) 252:114574. doi: 10.1016/j.ecoenv.2023.114574 36706525

[33] Shao Z Li D Chen J . Nanoplastics in simulated human lung fluids: Aggregation kinetics, theoretical model simulation, and effects on pulmonary bacteria. Environ Sci Technol. (2025) 59:16877–88. doi: 10.1021/acs.est.5c06466 40668842

[34] Ramsperger A Jasinski J Völkl M Witzmann T Meinhart M Jérôme V . Supposedly identical microplastic particles substantially differ in their material properties influencing particle-cell interactions and cellular responses. J Hazard Mater. (2022) 425:127961. doi: 10.1016/j.jhazmat.2021.127961 34986564

[35] Mognetti B Cecone C Fancello K Saraceni A Cottone E Bovolin P . Interaction of polystyrene nanoplastics with biomolecules and environmental pollutants: Effects on human hepatocytes. Int J Mol Sci. (2025) 26. doi: 10.3390/ijms26072899 40243532 PMC11988602

[36] Wieland S Ramsperger A Gross W Lehmann M Witzmann T Caspari A . Nominally identical microplastic models differ greatly in their particle-cell interactions. Nat Commun. (2024) 15:922. doi: 10.1038/s41467-024-45281-4 38297000 PMC10830523

[37] Codo AC Romero-Pichardo JE Wang Z Aufiero MA Lazarov T Saitz Rojas W . Polystyrene microplastic-induced pathophysiology is driven by disruption of efferocytosis. Immunity. (2026) 59:618–636.e611. doi: 10.1016/j.immuni.2026.01.009 41742421 PMC12948039

[38] Leslie HA van Velzen MJM Brandsma SH Vethaak AD Garcia-Vallejo JJ Lamoree MH . Discovery and quantification of plastic particle pollution in human blood. Environ Int. (2022) 163:107199. doi: 10.1016/j.envint.2022.107199 35367073

[39] Rauert C Charlton N Bagley A Dunlop SA Symeonides C Thomas KV . Assessing the efficacy of pyrolysis-gas chromatography-mass spectrometry for nanoplastic and microplastic analysis in human blood. Environ Sci Technol. (2025) 59:1984–94. doi: 10.1021/acs.est.4c12599 39851066 PMC11800385

[40] Kuntic M Kuntic I Cleppien D Pozzer A Nußbaum D Oelze M . Differential inflammation, oxidative stress and cardiovascular damage markers of nano- and micro-particle exposure in mice: Implications for human disease burden. Redox Biol. (2025) 83:103644. doi: 10.1016/j.redox.2025.103644 40319735 PMC12124686

[41] Huang Y Tang H Meng X Liu D Liu Y Chen B . Highly drug-loaded nanoaggregate microparticles for pulmonary delivery of cyclosporin A. Int J Nanomedicine. (2024) 19:7529–46. doi: 10.2147/ijn.S470134 39071501 PMC11283786

[42] Amato-Lourenço LF Carvalho-Oliveira R Júnior GR Dos Santos Galvão L Ando RA Mauad T . Presence of airborne microplastics in human lung tissue. J Hazard Mater. (2021) 416:126124. doi: 10.1016/j.jhazmat.2021.126124 34492918

[43] Qiu L Lu W Tu C Li X Zhang H Wang S . Evidence of microplastics in bronchoalveolar lavage fluid among never-smokers: A prospective case series. Environ Sci Technol. (2023) 57:2435–44. doi: 10.1021/acs.est.2c06880 36718593

[44] Wang S Lu W Cao Q Tu C Zhong C Qiu L . Microplastics in the lung tissues associated with blood test index. Toxics. (2023) 11. doi: 10.3390/toxics11090759 37755769 PMC10534820

[45] Uogintė I Vailionytė A Skapas M Bolanos D Bagurskienė E Gruslys V . New evidence of the presence of micro- and nanoplastic particles in bronchioalveolar lavage samples of clinical trial subjects. Heliyon. (2023) 9:e19665. doi: 10.1016/j.heliyon.2023.e19665 37809787 PMC10558899

[46] Liu S Wang C Yang Y Du Z Li L Zhang M . Microplastics in three types of human arteries detected by pyrolysis-gas chromatography/mass spectrometry (Py-GC/MS). J Hazard Mater. (2024) 469:133855. doi: 10.1016/j.jhazmat.2024.133855 38428296

[47] Eberhard T Casillas G Zarus GM Barr DB . Systematic review of microplastics and nanoplastics in indoor and outdoor air: identifying a framework and data needs for quantifying human inhalation exposures. J Expo Sci Environ Epidemiol. (2024) 34:185–96. doi: 10.1038/s41370-023-00634-x 38184724 PMC11142917

[48] Lu K Zhan D Fang Y Li L Chen G Chen S . Microplastics, potential threat to patients with lung diseases. Front Toxicol. (2022) 4:958414. doi: 10.3389/ftox.2022.958414 36245793 PMC9555848

[49] Anshika Jindal A Tomar D Mainuddin Kumar A . Microplastics: a cancer-causing agent for humans and prospects for identification using AI and modern approaches. Discov Oncol. (2025) 16:367. doi: 10.1007/s12672-024-01634-5 40111566 PMC11925826

[50] Ghio AJ Stewart M Sangani RG Pavlisko EN Roggli VL . Cigarette smoking decreases macrophage-dependent clearance to impact the biological effects of occupational and environmental particle exposures. Front Public Health. (2025) 13:1558723. doi: 10.3389/fpubh.2025.1558723 40270740 PMC12014686

[51] Ghio AJ Sangani RG Todd NW . The role of particle inhalation in idiopathic pulmonary fibrosis. Int J Mol Sci. (2025) 26. doi: 10.3390/ijms26178736 40943655 PMC12429018

[52] Chen C Luo L Zheng Y Ran L Li Z Guo Z . Evidence on microplastic exposure and respiratory health outcomes: a systematic review. Ther Adv Respir Dis. (2026) 20:17534666261465486. doi: 10.1177/17534666261465486 42460774 PMC13376494

[53] Paplińska-Goryca M Misiukiewicz-Stępień P Wróbel M Mycroft-Rzeszotarska K Adamska D Rachowka J . The impaired response of nasal epithelial cells to microplastic stimulation in asthma and COPD. Sci Rep. (2025) 15:4242. doi: 10.1038/s41598-025-87242-x 39905077 PMC11794662

[54] Merkley SD Moss HC Goodfellow SM Ling CL Meyer-Hagen JL Weaver J . Polystyrene microplastics induce an immunometabolic active state in macrophages. Cell Biol Toxicol. (2022) 38:31–41. doi: 10.1007/s10565-021-09616-x 34021430 PMC8606615

[55] Li Y Shi T Li X Sun H Xia X Ji X . Inhaled tire-wear microplastic particles induced pulmonary fibrotic injury via epithelial cytoskeleton rearrangement. Environ Int. (2022) 164:107257. doi: 10.1016/j.envint.2022.107257 35486965

[56] Tomonaga T Higashi H Izumi H Nishida C Kawai N Sato K . Investigation of pulmonary inflammatory responses following intratracheal instillation of and inhalation exposure to polypropylene microplastics. Part Fibre Toxicol. (2024) 21:29. doi: 10.1186/s12989-024-00592-8 39107780 PMC11301944

[57] Li X Sun Q Li J Ma J Xu R Fan J . Inhalation and deposition characteristics of respiratory ultrafine and elongated mineral particles in the all-in-one human respiratory system: A CFD-based study. J Hazard Mater. (2026) 514:142637. doi: 10.1016/j.jhazmat.2026.142637 42378760

[58] Song MK Eun Park J Ryu SH Baek YW Kim YH Im Kim D . Biodistribution and respiratory toxicity of chloromethylisothiazolinone/methylisothiazolinone following intranasal and intratracheal administration. Environ Int. (2022) 170:107643. doi: 10.1016/j.envint.2022.107643 36403329

[59] Bhagat K Barrios AC Rajwade K Kumar A Oswald J Apul O . Aging of microplastics increases their adsorption affinity towards organic contaminants. Chemosphere. (2022) 298:134238. doi: 10.1016/j.chemosphere.2022.134238 35276106

[60] Dou JY Liu S Wang CY Dai X Lian LH Cui ZY . Microplastics and nanoplastics, emerging pollutants, increased the risk of pulmonary fibrosis *in vivo* and *in vitro*: a comparative evaluation of their potential toxicity effects with different polymers and size. Toxicology. (2026) 519:154304. doi: 10.1016/j.tox.2025.154304 41075967

[61] Zhang T Yang S Ge Y Wan X Zhu Y Li J . Polystyrene nanoplastics induce lung injury via activating oxidative stress: Molecular insights from bioinformatics analysis. Nanomaterials (Basel). (2022) 12. doi: 10.3390/nano12193507 36234635 PMC9565894

[62] Danso IK Woo JH Lee K . Pulmonary toxicity of polystyrene, polypropylene, and polyvinyl chloride microplastics in mice. Molecules. (2022) 27. doi: 10.3390/molecules27227926 36432032 PMC9694469

[63] Lu H Guo T Zhang Y Liu D Hou L Ma C . Endoplasmic reticulum stress-induced NLRP3 inflammasome activation as a novel mechanism of polystyrene microplastics (PS-MPs)-induced pulmonary inflammation in chickens. J Zhejiang Univ-Sci B. (2024) 25:233–43. doi: 10.1631/jzus.B2300409 38453637 PMC10918412

[64] Peng M Vercauteren M Grootaert C Rajkovic A Boon N Janssen C . Cellular and bioenergetic effects of polystyrene microplastic in function of cell type, differentiation status and post-exposure time. Environ pollut. (2023) 337:122550. doi: 10.1016/j.envpol.2023.122550 37716692

[65] Kecorius S Madueño L Birmili W Löndahl J Plauškaitė K Byčenkienė S . Urban pollution impact assessment in six Lithuanian cities with a focus on road traffic emissions - integrated framework for environmental health studies. J Hazard Mater. (2025) 497:139725. doi: 10.1016/j.jhazmat.2025.139725 40907320

[66] Lin K Wallis C Wong EM Edwards P Cole A Van Winkle L . Heterogeneous deposition of regular and mentholated little cigar smoke in the lungs of Sprague-Dawley rats. Part Fibre Toxicol. (2023) 20:42. doi: 10.1186/s12989-023-00554-6 37932763 PMC10626780

[67] Aurell J Holder AL Gullett BK Sowers TD Weinstein J Kariher P . Gas and particle emissions from rifle and pistol firing. J Hazard Mater. (2024) 476:135196. doi: 10.1016/j.jhazmat.2024.135196 39018594 PMC11459210

[68] Tang X Lu L Sun Y Li A Su W Cao J . Unveiling the pulmonary toxicity of polystyrene nanoplastics: A hierarchical oxidative stress mechanism driving acute-subacute lung injury. Res (Wash D C). (2025) 8:995. doi: 10.34133/research.0995 41292701 PMC12641161

[69] Wei Y Wu Y Zhu D Zhang J Li X Wang M . Polystyrene-nanoplastics-induced unfolded protein response in monocyte-derived macrophages mediates pulmonary fibrosis via oxidative-stress-dependent IL-6 secretion. J Hazard Mater. (2026) 506:141476. doi: 10.1016/j.jhazmat.2026.141476 41747702

[70] Zhou H Liu Y Ren Y Jiang N Sun A Wu S . Polystyrene microplastics induce radiotherapy resistance in lung cancer by suppressing ferroptosis through NF-κB activation. Antioxid Redox Signal. (2026) 44:118–33. doi: 10.1177/15230864251389969 41192841

[71] Wu Y Yao Y Wu C He R Huang Z Zhang C . Inhalation exposure to cationic nanoplastic induces ferroptosis in the lung by perturbing core circadian transcription factors Bmal1. J Hazard Mater. (2025) 495:139048. doi: 10.1016/j.jhazmat.2025.139048 40578193

[72] Wang Y Wei J Deng H Zheng L Yang H Lv X . The role of Nrf2 in pulmonary fibrosis: Molecular mechanisms and treatment approaches. Antioxidants (Basel). (2022) 11. doi: 10.3390/antiox11091685 36139759 PMC9495339

[73] Sakai T Takagaki H Yamagiwa N Ui M Hatta S Imai J . Effects of the cytoplasm and mitochondrial specific hydroxyl radical scavengers TA293 and mitoTA293 in bleomycin-induced pulmonary fibrosis model mice. Antioxidants (Basel). (2021) 10. doi: 10.3390/antiox10091398 34573030 PMC8469049

[74] Halimu G Zhang Q Liu L Zhang Z Wang X Gu W . Toxic effects of nanoplastics with different sizes and surface charges on epithelial-to-mesenchymal transition in A549 cells and the potential toxicological mechanism. J Hazard Mater. (2022) 430:128485. doi: 10.1016/j.jhazmat.2022.128485 35739668

[75] Jeon S Jeon JH Jeong J Kim G Lee S Kim S . Size- and oxidative potential-dependent toxicity of environmentally relevant expanded polystyrene styrofoam microplastics to macrophages. J Hazard Mater. (2023) 459:132295. doi: 10.1016/j.jhazmat.2023.132295 37597397

[76] Tanveer M Mansha N Nimra A Khawar MB Afzal A Afzal H . Microplastics: Unraveling the signaling pathways involved in reproductive health. Environ Sci pollut Res Int. (2023) 30:95077–85. doi: 10.1007/s11356-023-29273-3 37584803

[77] Wang Z Wang N Jiang S Shi R Wang R Wu W . Polystyrene nanoplastics induced lung injury in mice: Insights into lung metabolic disorders. Ecotoxicol Environ Saf. (2025) 303:119060. doi: 10.1016/j.ecoenv.2025.119060 40967017

[78] Jeon S Jeon JH Kim G Yang SI Cho WS . Environmentally relevant aged nanoplastics amplify oxidative stress-associated inhalation toxicity and delay lung clearance. Redox Biol. (2026) 95:104281. doi: 10.1016/j.redox.2026.104281 42372706 PMC13331772

[79] Wu Y Yao Y Bai H Shimizu K Li R Zhang C . Investigation of pulmonary toxicity evaluation on mice exposed to polystyrene nanoplastics: The potential protective role of the antioxidant N-acetylcysteine. Sci Total Environ. (2023) 855:158851. doi: 10.1016/j.scitotenv.2022.158851 36155047

[80] Zhu X Chu Z Xie P Chen D Liu X Chen H . Size-dependent toxicity of polystyrene microplastics in lung cells: An *in vivo* and *in vitro* study. Ecotoxicol Environ Saf. (2025) 307:119456. doi: 10.1016/j.ecoenv.2025.119456 41289762

[81] Han Z Wang L Li X Dai Y Guan X Su Z . Repeated inhalation exposure to polystyrene nanoplastics induced sustained pulmonary injury and fibrosis in mice. Ecotoxicol Environ Saf. (2026) 313:120007. doi: 10.1016/j.ecoenv.2026.120007 41832876

[82] Cao J Xu R Geng Y Xu S Guo M . Exposure to polystyrene microplastics triggers lung injury via targeting toll-like receptor 2 and activation of the NF-κB signal in mice. Environ pollut. (2023) 320:121068. doi: 10.1016/j.envpol.2023.121068 36641069

[83] Yang S Zhang T Ge Y Yin L Pu Y Liang G . Inhalation exposure to polystyrene nanoplastics induces chronic obstructive pulmonary disease-like lung injury in mice through multi-dimensional assessment. Environ pollut. (2024) 347:123633. doi: 10.1016/j.envpol.2024.123633 38423272

[84] Wu Q Liu C Liu D Wang Y Qi H Liu X . Polystyrene nanoplastics-induced lung apoptosis and ferroptosis via ROS-dependent endoplasmic reticulum stress. Sci Total Environ. (2024) 912:169260. doi: 10.1016/j.scitotenv.2023.169260 38086481

[85] Jin S Yoon SJ Jung NY Lee WS Jeong J Park YJ . Antioxidants prevent particulate matter-induced senescence of lung fibroblasts. Heliyon. (2023) 9:e14179. doi: 10.1016/j.heliyon.2023.e14179 36915477 PMC10006845

[86] Wu Y Wang J Zhao T Sun M Xu M Che S . Polystyrenenanoplastics lead to ferroptosis in the lungs. J Adv Res. (2024) 56:31–41. doi: 10.1016/j.jare.2023.03.003 36933884 PMC10834790

[87] Yang S Zhang T Ge Y Cheng Y Yin L Pu Y . Ferritinophagy mediated by oxidative stress-driven mitochondrial damage is involved in the polystyrene nanoparticles-induced ferroptosis of lung injury. ACS Nano. (2023) 17:24988–5004. doi: 10.1021/acsnano.3c07255 38086097

[88] Ling H Xiao H Luo T Lin H Deng J . Role of ferroptosis in regulating the epithelial-mesenchymal transition in pulmonary fibrosis. Biomedicines. (2023) 11. doi: 10.3390/biomedicines11010163 36672671 PMC9856078

[89] Hu Y Huang Y Zong L Lin J Liu X Ning S . Emerging roles of ferroptosis in pulmonary fibrosis: current perspectives, opportunities and challenges. Cell Death Discov. (2024) 10:301. doi: 10.1038/s41420-024-02078-0 38914560 PMC11196712

[90] Yao M Liu Z Zhao W Song S Huang X Wang Y . Ferroptosis in idiopathic pulmonary fibrosis: Mechanisms, impact, and therapeutic opportunities. Front Immunol. (2025) 16:1567994. doi: 10.3389/fimmu.2025.1567994 40469306 PMC12133551

[91] Zhang J Zhang L Chen Y Fang X Li B Mo C . The role of cGAS-STING signaling in pulmonary fibrosis and its therapeutic potential. Front Immunol. (2023) 14:1273248. doi: 10.3389/fimmu.2023.1273248 37965345 PMC10642193

[92] Park M Park S Choi Y Cho YL Kim MJ Park YJ . The mechanism underlying correlation of particulate matter-induced ferroptosis with inflammasome activation and iron accumulation in macrophages. Cell Death Discov. (2024) 10:144. doi: 10.1038/s41420-024-01874-y 38491062 PMC10943117

[93] Song H Peng L Yang Y Sun K Chu Y Deng M . Dual targeting of NCF1 and NLRP3 by roburic acid orchestrates redox homeostasis and inhibits macrophage death in septic lung injury. Redox Biol. (2026) 93:104161. doi: 10.1016/j.redox.2026.104161 41980460 PMC13092860

[94] Lage SL Amaral EP Hilligan KL Laidlaw E Rupert A Namasivayan S . Persistent oxidative stress and inflammasome activation in CD14(high)CD16(-) monocytes from COVID-19 patients. Front Immunol. (2021) 12:799558. doi: 10.3389/fimmu.2021.799558 35095880 PMC8795739

[95] Liu CC Lin JH Hsu TW Wang CY Hsu HS . Inhibition of the 4-hydroxynonenal-regulated JNK/c-Jun pathway improves bleomycin-induced lung fibrosis. BioMed J. (2026) 49:100916. doi: 10.1016/j.bj.2025.100916 41076274 PMC13226823

[96] Liu J Xu F Guo M Gao D Song Y . Nasal instillation of polystyrene nanoplastics induce lung injury via mitochondrial DNA release and activation of the cyclic GMP-AMP synthase-stimulator of interferon genes-signaling cascade. Sci Total Environ. (2024) 948:174674. doi: 10.1016/j.scitotenv.2024.174674 39002594

[97] Sul OJ Kim JH Lee T Seo KW Cha HJ Kwon B . GSPE protects against bleomycin-induced pulmonary fibrosis in mice via ameliorating epithelial apoptosis through inhibition of oxidative stress. Oxid Med Cell Longev. (2022) 2022:8200189. doi: 10.1155/2022/8200189 35355866 PMC8958066

[98] Fresilli S Belletti A Labanca R Monti G Schultz MJ Corbo F . Nebulized heparin in adults with acute respiratory failure: a meta-analysis of randomized trials. Crit Care Med. (2026) 54:1755–66. doi: 10.1097/ccm.0000000000007161 42148826

[99] Chen Y Zhang Y Zhang Y Lv S Zhang X Mohsen M . Polystyrene nanoplastics and benzo(a)pyrene synergistically induce lung fibrosis and inflammation via relaxin signalling in mice. Commun Biol. (2026) 9. doi: 10.1038/s42003-026-09872-9 41862678 PMC13168281

[100] Woo JH Song MK Baek SH Park CJ Kim JW Kim B . Polystyrene particles induces asthma-like Th2-mediated lung injury through IL-33 secretion. Environ Int. (2025) 203:109772. doi: 10.1016/j.envint.2025.109772 40926454

[101] Liu H Wang D Li J Wang Y Liang Z Chen X . Gasdermin D-dependent macrophage pyroptosis mediates polystyrene microplastics-induced pulmonary fibrosis. Int Immunopharmacol. (2026) 172:116189. doi: 10.1016/j.intimp.2026.116189 41539003

[102] Alijagic A Hedbrant A Persson A Larsson M Engwall M Särndahl E . NLRP3 inflammasome as a sensor of micro- and nanoplastics immunotoxicity. Front Immunol. (2023) 14:1178434. doi: 10.3389/fimmu.2023.1178434 37143682 PMC10151538

[103] Bai Y Zhan X Zhu Q Ji X Lu Y Gao Y . ATG16L1 restrains macrophage NLRP3 activation and alveolar epithelial cell injury during septic lung injury. Clin Transl Med. (2025) 15:e70289. doi: 10.1002/ctm2.70289 40211890 PMC11986372

[104] Wei YY Chen TT Zhang DW Zhang Y Li F Ding YC . Microplastics exacerbate ferroptosis via mitochondrial reactive oxygen species-mediated autophagy in chronic obstructive pulmonary disease. Autophagy. (2025) 21:1717–43. doi: 10.1080/15548627.2025.2481126 40114310 PMC12283003

[105] Li Z Yao YX Lu X Peng K He YZ Liu ZB . Short-term respiratory cadmium exposure partially activates pulmonary NLRP3 inflammasome by inducing ferroptosis in mice. Ecotoxicol Environ Saf. (2024) 285:117106. doi: 10.1016/j.ecoenv.2024.117106 39326353

[106] Wang Y Duan H Zhang J Wang Q Peng T Ye X . YAP1 protects against PM2.5-induced lung toxicity by suppressing pyroptosis and ferroptosis. Ecotoxicol Environ Saf. (2023) 253:114708. doi: 10.1016/j.ecoenv.2023.114708 36863160

[107] Wu H Xu T Chen T Liu J Xu S . Oxidative stress mediated by the TLR4/NOX2 signalling axis is involved in polystyrene microplastic-induced uterine fibrosis in mice. Sci Total Environ. (2022) 838:155825. doi: 10.1016/j.scitotenv.2022.155825 35597360

[108] Somanader DVN Zhao P Widdop RE Samuel CS . The involvement of the Wnt/β-catenin signaling cascade in fibrosis progression and its therapeutic targeting by relaxin. Biochem Pharmacol. (2024) 223:116130. doi: 10.1016/j.bcp.2024.116130 38490518

[109] Ajoolabady A Pratico D Bahijri S Tuomilehto J Uversky VN Ren J . Hallmarks of cellular senescence: biology, mechanisms, regulations. Exp Mol Med. (2025) 57:1482–91. doi: 10.1038/s12276-025-01480-7 40634753 PMC12322015

[110] Luo H Xiao T Sun X Song Y Shi W Lu K . The regulation of circRNA_kif26b on alveolar epithelial cell senescence via miR-346-3p is involved in microplastics-induced lung injuries. Sci Total Environ. (2023) 882:163512. doi: 10.1016/j.scitotenv.2023.163512 37084911

[111] Jin W Zhang W Tang H Wang P Zhang Y Liu S . Microplastics exposure causes the senescence of human lung epithelial cells and mouse lungs by inducing ROS signaling. Environ Int. (2024) 185:108489. doi: 10.1016/j.envint.2024.108489 38367553

[112] Roger I Milara J Belhadj N Cortijo J . Senescence alterations in pulmonary hypertension. Cells. (2021) 10. doi: 10.3390/cells10123456 34943963 PMC8700581

[113] Li YQ An XL Jin FY Bai YF Li T Yang XY . ISRIB inhibits the senescence of type II pulmonary epithelial cells to alleviate pulmonary fibrosis induced by silica in mice. Ecotoxicol Environ Saf. (2023) 264:115410. doi: 10.1016/j.ecoenv.2023.115410 37647802

[114] Cui X Wang Y Fu J . DNA damage response and cell fate decisions across the lifespan: from fetal development to age-related respiratory diseases. Cell Biosci. (2025) 15:114. doi: 10.1186/s13578-025-01442-6 40753255 PMC12317523

[115] Wu D Zhang M Bao TT Lan H . Long-term exposure to polystyrene microplastics triggers premature testicular aging. Part Fibre Toxicol. (2023) 20:35. doi: 10.1186/s12989-023-00546-6 37641072 PMC10463354

[116] Li SR Kang NN Wang RR Li MD Chen LH Zhou P . ALKBH5 SUMOylation-mediated FBXW7 m6A modification regulates alveolar cells senescence during 1-nitropyrene-induced pulmonary fibrosis. J Hazard Mater. (2024) 468:133704. doi: 10.1016/j.jhazmat.2024.133704 38364577

[117] Li Y Chen R Wu J Xue X Liu T Peng G . Salvianolic acid B protects against pulmonary fibrosis by attenuating stimulating protein 1-mediated macrophage and alveolar type 2 cell senescence. Phytother Res. (2024) 38:620–35. doi: 10.1002/ptr.8070 37953063

[118] Alqahtani S Alqahtani T Venkatesan K Sivadasan D Ahmed R Sirag N . SASP modulation for cellular rejuvenation and tissue homeostasis: therapeutic strategies and molecular insights. Cells. (2025) 14. doi: 10.3390/cells14080608 40277933 PMC12025513

[119] Zhang X Zhao W Hu X Shen Y Gan Y Zou J . Activation of Sirt1 protects from single-walled carbon nanotubes-induced pulmonary fibrosis by inhibiting alveolar macrophage senescence. Ecotoxicol Environ Saf. (2025) 302:118629. doi: 10.1016/j.ecoenv.2025.118629 40627914

[120] Xiao J Li HS Satyanarayanan SK Leung SL Yuan Q Wang Y . Advancements in targeting macrophage senescence for age-associated conditions. Aging Dis. (2024) 16:2201–36. doi: 10.14336/ad.2024.0720 39500353 PMC12221398

[121] Schafer MJ White TA Iijima K Haak AJ Ligresti G Atkinson EJ . Cellular senescence mediates fibrotic pulmonary disease. Nat Commun. (2017) 8:14532. doi: 10.1038/ncomms14532 28230051 PMC5331226

[122] Liu Z Meng L Yang B Hu Y Yang Z Liu Y . Zanubrutinib mitigates pulmonary fibrosis in aged mice by targeting the Wnt/β-catenin signaling pathway. Biochem Pharmacol. (2026) 243:117428. doi: 10.1016/j.bcp.2025.117428 41110481

[123] Du Y Zhang J Zhuo J Chen J Liu D Li Y . 5-methoxyindole-3-carboxaldehyde attenuates alveolar type II epithelial cell senescence induced by exposure to polystyrene microplastics in pulmonary fibrosis via PI3K/AKT/mTOR signaling pathway. Ecotoxicol Environ Saf. (2026) 310:119782. doi: 10.1016/j.ecoenv.2026.119782 41610587

[124] Dubey R Baby S Singh A Singh S Singh N Goswami P . Polystyrene microplastics activate noncanonical TGF-β signaling and metabolomic reprogramming to promote epithelial-mesenchymal transition and fibrosis in the kidney. ACS Omega. (2026) 11:33395–415. doi: 10.1021/acsomega.5c10834 42326637 PMC13280856

[125] Wei P Xie Y Abel PW Huang Y Ma Q Li L . Transforming growth factor (TGF)-β1-induced miR-133a inhibits myofibroblast differentiation and pulmonary fibrosis. Cell Death Dis. (2019) 10:670. doi: 10.1038/s41419-019-1873-x 31511493 PMC6739313

[126] Hu Y Wang Q Yu J Zhou Q Deng Y Liu J . Tartrate-resistant acid phosphatase 5 promotes pulmonary fibrosis by modulating β-catenin signaling. Nat Commun. (2022) 13:114. doi: 10.1038/s41467-021-27684-9 35013220 PMC8748833

[127] Liao W Ang Y Kee ACL Lim V Lim AYH Chai CLL . Calcaratarin D, a labdane diterpenoid, attenuates bleomycin-induced pulmonary fibrosis by blocking Wnt/β-catenin signaling pathway. Pharmacol Res. (2025) 216:107756. doi: 10.1016/j.phrs.2025.107756 40311955

[128] Lewis A Sánchez S Berti G Pan-Castillo B Nijhuis A Mehta S . Small-molecule Wnt inhibitors are a potential novel therapy for intestinal fibrosis in Crohns disease. Clin Sci (Lond). (2022) 136:1405–23. doi: 10.1042/cs20210889 36156078 PMC9583737

[129] Kim DE Shin SB Kim CH Kim YB Oh HJ Yim H . PLK1-mediated phosphorylation of β-catenin enhances its stability and transcriptional activity for extracellular matrix remodeling in metastatic NSCLC. Theranostics. (2023) 13:1198–216. doi: 10.7150/thno.79318 36793862 PMC9925311

[130] Guo C Ai S Chen J . Tanshinone IIA attenuates pulmonary fibrosis via dual inhibition of JNK and Smad signaling. Antioxidants (Basel). (2026) 15. doi: 10.3390/antiox15070836 42510567 PMC13405772

[131] Enomoto Y Katsura H Fujimura T Ogata A Baba S Yamaoka A . Autocrine TGF-β-positive feedback in profibrotic AT2-lineage cells plays a crucial role in non-inflammatory lung fibrogenesis. Nat Commun. (2023) 14:4956. doi: 10.1038/s41467-023-40617-y 37653024 PMC10471635

[132] Traversa A Mari E Pontecorvi P Gerini G Romano E Megiorni F . Polyethylene micro/nanoplastics exposure induces epithelial-mesenchymal transition in human bronchial and alveolar epithelial cells. Int J Mol Sci. (2024) 25. doi: 10.3390/ijms251810168 39337653 PMC11432389

[133] Itano J Taniguchi A Senoo S Asada N Gion Y Egusa Y . Neuropeptide Y antagonizes development of pulmonary fibrosis through IL-1β inhibition. Am J Respir Cell Mol Biol. (2022) 67:654–65. doi: 10.1165/rcmb.2021-0542OC 36122332

[134] Lei M Chen G . Integration of mechanics and immunology: Perspective for understanding fibrotic disease mechanisms and innovating therapeutic strategies. Acta Biomater. (2025) 199:35–49. doi: 10.1016/j.actbio.2025.05.011 40324516

[135] Sun X Xiao T Qin J Song Y Lu K Ding R . Mechanism of circRNA_SMG6 mediating lung macrophage ECM degradation via miR-570-3p in microplastics-induced emphysema. Environ Int. (2024) 187:108701. doi: 10.1016/j.envint.2024.108701 38685156

[136] Zhao P Yin S Tao H . ECM stiffness and epigenetics in organ fibrosis. Trends Pharmacol Sci. (2026) 47:720–5. doi: 10.1016/j.tips.2026.05.009 42270465

[137] Huang D Chen H Shen M Tao J Chen S Yin L . Recent advances on the transport of microplastics/nanoplastics in abiotic and biotic compartments. J Hazard Mater. (2022) 438:129515. doi: 10.1016/j.jhazmat.2022.129515 35816806

[138] Carriera F Di Fiore C Avino P . Trojan horse effects of microplastics: a mini-review about their role as a vector of organic and inorganic compounds in several matrices. AIMS Environ Sci. (2023) 10:732–42. doi: 10.3934/environsci.2023040

[139] Alaraby M Abass D Villacorta A Hernández A Marcos R . Antagonistic *in vivo* interaction of polystyrene nanoplastics and silver compounds. A study using Drosophila. Sci Total Environ. (2022) 842:156923. doi: 10.1016/j.scitotenv.2022.156923 35753490

[140] Epeslidou E Scott JS de Klein B Cudia JT Melgert B Prekovic S . Microplastics as environmental modifiers of lung disease. EMBO Mol Med. (2026) 18:381–95. doi: 10.1038/s44321-025-00353-w 41381807 PMC12905266

[141] Tong X Fu X Yu G Qu H Zou H Song R . Polystyrene exacerbates cadmium-induced mitochondrial damage to lung by blocking autophagy in mice. Environ Toxicol. (2023) 38:1775–85. doi: 10.1002/tox.23804 37022104

[142] Ji Y Wang Y Liu Y Yang Z Liu B Liu W . Nanoplastics and benzo[a]pyrene co-pollution aggravates pulmonary ferroptosis via endoplasmic reticulum stress-triggered IRE1α liquid-liquid phase separation. Ecotoxicol Environ Saf. (2026) 322:120347. doi: 10.1016/j.ecoenv.2026.120347 42259117

[143] Cedergreen N . Quantifying synergy: a systematic review of mixture toxicity studies within environmental toxicology. PloS One. (2014) 9:e96580. doi: 10.1371/journal.pone.0096580 24794244 PMC4008607

[144] Martin O Scholze M Ermler S McPhie J Bopp SK Kienzler A . Ten years of research on synergisms and antagonisms in chemical mixtures: A systematic review and quantitative reappraisal of mixture studies. Environ Int. (2021) 146:106206. doi: 10.1016/j.envint.2020.106206 33120228

[145] Koelmans AA Bakir A Burton GA Janssen CR . Microplastic as a vector for chemicals in the aquatic environment: Critical review and model-supported reinterpretation of empirical studies. Environ Sci Technol. (2016) 50:3315–26. doi: 10.1021/acs.est.5b06069 26946978 PMC6863595

[146] Shi Q Tang J Wang L Liu R Giesy JP . Combined cytotoxicity of polystyrene nanoplastics and phthalate esters on human lung epithelial A549 cells and its mechanism. Ecotoxicol Environ Saf. (2021) 213:112041. doi: 10.1016/j.ecoenv.2021.112041 33601174

[147] Richeldi L du Bois RM Raghu G Azuma A Brown KK Costabel U . Efficacy and safety of nintedanib in idiopathic pulmonary fibrosis. N Engl J Med. (2014) 370:2071–82. doi: 10.1056/NEJMoa1402584 24836310

[148] King TE Jr. Bradford WZ Castro-Bernardini S Fagan EA Glaspole I Glassberg MK . A phase 3 trial of pirfenidone in patients with idiopathic pulmonary fibrosis. N Engl J Med. (2014) 370:2083–92. doi: 10.1056/NEJMoa1402582 24836312

[149] Bai L Wang J Wang X Wang J Zeng W Pang J . Combined therapy with pirfenidone and nintedanib counteracts fibrotic silicosis in mice. Br J Pharmacol. (2025) 182:1143–63. doi: 10.1111/bph.17390 39546810

[150] Andrade da Silva LH Vieira JB Cabral MR Antunes MA Lee D Cruz FF . Development of nintedanib nanosuspension for inhaled treatment of experimental silicosis. Bioeng Transl Med. (2023) 8:e10401. doi: 10.1002/btm2.10401 36925690 PMC10013831

[151] Justice JN Nambiar AM Tchkonia T LeBrasseur NK Pascual R Hashmi SK . Senolytics in idiopathic pulmonary fibrosis: Results from a first-in-human, open-label, pilot study. EBioMedicine. (2019) 40:554–63. doi: 10.1016/j.ebiom.2018.12.052 30616998 PMC6412088

[152] Nambiar A Kellogg D Justice J Goros M Gelfond J Pascual R . Senolytics dasatinib and quercetin in idiopathic pulmonary fibrosis: results of a phase I, single-blind, single-center, randomized, placebo-controlled pilot trial on feasibility and tolerability. EBioMedicine. (2023) 90:104481. doi: 10.1016/j.ebiom.2023.104481 36857968 PMC10006434

[153] Hong DS Kang YK Borad M Sachdev J Ejadi S Lim HY . Phase 1 study of MRX34, a liposomal miR-34a mimic, in patients with advanced solid tumours. Br J Cancer. (2020) 122:1630–7. doi: 10.1038/s41416-020-0802-1 32238921 PMC7251107

[154] Gale DP Gross O Wang F Esteban de la Rosa RJ Hall M Sayer JA . A randomized controlled clinical trial testing effects of lademirsen on kidney function decline in adults with Alport syndrome. Clin J Am Soc Nephrol. (2024) 19:995–1004. doi: 10.2215/cjn.0000000000000458 38829703 PMC11321738

[155] Gallant-Behm CL Piper J Lynch JM Seto AG Hong SJ Mustoe TA . A microRNA-29 mimic (Remlarsen) represses extracellular matrix expression and fibroplasia in the skin. J Invest Dermatol. (2019) 139:1073–81. doi: 10.1016/j.jid.2018.11.007 30472058

[156] Winkle M El-Daly SM Fabbri M Calin GA . Noncoding RNA therapeutics - challenges and potential solutions. Nat Rev Drug Discov. (2021) 20:629–51. doi: 10.1038/s41573-021-00219-z 34145432 PMC8212082

[157] Cui H Yang W Cui Y Qi L Jiang X Li M . Adverse effects of pristine and aged polystyrene microplastics in mice and their Nrf2-mediated defense mechanisms with tissue specificity. Environ Sci pollut Res Int. (2023) 30:39894–906. doi: 10.1007/s11356-022-24918-1 36602732

